# Compositional Causal Identification from Imperfect or Disturbing Observations

**DOI:** 10.3390/e27070732

**Published:** 2025-07-08

**Authors:** Isaac Friend, Aleks Kissinger, Robert W. Spekkens, Elie Wolfe

**Affiliations:** 1 Department of Computer Science, University of Oxford, Oxford OX1 3QD, UK; aleks.kissinger@cs.ox.ac.uk; 2 Perimeter Institute for Theoretical Physics, 31 Caroline Street North, Waterloo, ON N2L 2Y5, Canada; rspekkens@perimeterinstitute.ca (R.W.S.); ewolfe@perimeterinstitute.ca (E.W.)

**Keywords:** causal identification, process theories, causal Bayesian networks, directed acyclic graphs, acyclic directed mixed graphs, string diagrams

## Abstract

The usual inputs for a causal identification task are a graph representing qualitative causal hypotheses and a joint probability distribution for some of the causal model’s variables when they are observed rather than intervened on. Alternatively, the available probabilities sometimes come from a combination of passive observations and controlled experiments. It also makes sense, however, to consider causal identification with data collected via schemes more generic than (perfect) passive observation or perfect controlled experiments. For example, observation procedures may be noisy, may disturb the variables, or may yield only coarse-grained specification of the variables’ values. In this work, we investigate identification of causal quantities when the probabilities available for inference are the probabilities of outcomes of these more generic schemes. Using process theories (aka symmetric monoidal categories), we formulate graphical causal models as second-order processes that respond to such data collection instruments. We pose the causal identification problem relative to arbitrary sets of available instruments. Perfect passive observation instruments—those that produce the usual observational probabilities used in causal inference—satisfy an abstract process-theoretic property called *marginal informational completeness*. This property also holds for other (sets of) instruments. The main finding is that in the case of Markovian models, as long as the available instruments satisfy this property, the probabilities they produce suffice for identification of interventional quantities, just as those produced by perfect passive observations do. This finding sharpens the distinction between the Markovianity of a causal model and that of a probability distribution, suggesting a more extensive line of investigation of causal inference within a process-theoretic framework.

## 1. Introduction

One use of causal graphical models is to formulate and solve problems of *identification of causal effects*—or *causal identification*—in which hypotheses about the existence and direction of causal relationships between variables are used together with observational data to infer the effects of interventions. The interventions with effects to be computed may be either “atomic” interventions, represented with Pearl’s “do” operator [1], or more complicated ones involving randomness or dependence on other variables in a model [2]. Similarly, the data available for inference may be simply the joint probabilities of outcomes of what we will call *perfect passive observation*, or may be outcome probabilities associated with various kinds of combinations of perfect passive observation via controlled experiments [3]. In this work, we define graphical causal models in a way that is equivalent to standard ones but is designed to support reasoning about quite general classes of interactions between an agent and a causal data-generating process. This framework allows us to pose and solve new kinds of causal identification problems based on these more general types of probing schemes. We demonstrate the application of such a framework in the case of Markovian models, i.e., those in which every variable can be observed at least in some way. It is well known that for a Markovian causal model associated with a directed acyclic graph, all the model’s parameters, and hence essentially all interventional quantities, are identifiable from the graph and the joint distribution of the model’s variables under perfect passive observation. Identifiability seems to depend on the existence of a certain factorization of that distribution. We consider probing schemes that may yield only partial information about variables’ values and that may also disturb those values. The outcome distributions for these schemes cannot generally be factored in the same way as distributions obtained via perfect passive observations; however, if the probing scheme satisfies certain abstract criteria, then it may serve as well as ordinary perfect passive observations for the purpose of identification in Markovian models.

In a graphical causal model as we define it, each node in a directed graph corresponds to an *intervention locus* where a variable “arrives” and then “leaves”, with the possibility for probing or manipulation in between. A generic interaction with a variable at a locus is represented by an *instrument*, examples of which include a perfect passive observation, which reports the value of a variable without changing it, and a *surgical* intervention, which discards the incoming value and sets the outgoing value. With all types of probing schemes represented by the same sort of mathematical entity, we can pose different kinds of causal identification problems by declaring different kinds of interaction to be available as means of gathering data for inference. Thus, we can ask about identification of causal quantities from statistics generated under combinations of passive observation and controlled experiments [4], or more generally under probing schemes that cannot obviously be classified as purely observational or purely experimental. Formulating and solving identification problems using these more generic probing schemes is the main contribution of the present paper.

The mathematical technology used in this paper is that of *process theories*, which are categories for composing input–output processes in sequence and in parallel. These categories arise in logic, computation, physics, and topology [5]. They admit graphical calculi of *string diagrams*, which we use to represent and calculate with processes. The present article builds on an approach to causal inference [6,7] in which the qualitative causal hypotheses in a directed acyclic graph specify a morphism in a *syntactic* process theory; then, a concrete data-generating process conforming to those hypotheses is a functorial interpretation of that morphism in a *semantic* process theory of matrices (the first of these cited works addresses causal identification only with ordinary observational data, i.e., data from perfect passive observations; the second does not discuss identification at all). This article also shares technical infrastructure with [8] and the conference paper [9]. It generalizes a result from [9] (specifically, it generalizes the $\mathrm{Mat}[R_{+}]$ case of Proposition 28 of [9] to graphs with arbitrarily many nodes and to instruments that may not be ∘-separable), but uses a different technique and is not concerned with the parallel treatment of classical and quantum causal models.

## 2. Process Theories, Bayesian Networks, and Causal Identification

A Bayesian network of the most basic kind consists of a directed acyclic graph and a probability distribution that is Markov-compatible with that graph. For example, a joint distribution P(ABCDE) is Markov-compatible with the graph
(1)
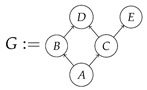

precisely when

(2) P(ABCDE)=P(A)P(B|A)P(C|A)P(D|BC)P(E|C).

### 2.1. Process Theories

We represent both the distribution and the factorization implied by the graph using process-theoretic formalism. A *process theory* is a certain kind of category used for representing the parallel and sequential composition of input–output processes (e.g., matrices). It consists of objects, called *system-types* (e.g., integers indicating cardinalities of variables), and morphisms between system-types, called *processes*, with the ordinary composition ∘ viewed as sequential composition of processes, and another operation ⊗ for composing system-types and processes in parallel.

One of the system-types, say *I*, is a unit for ⊗ (i.e., for any system-type *A*, A⊗I=A=I⊗A) and represents “empty” systems. It appears as the domain of processes “with no input” and as the codomain of processes “with no output”. Processes of the form ρ:I→A are called *states*, and processes of the form π:A→I are called *effects* (the term “effect” comes from quantum theory, where it refers to a physical effect resulting from a measurement [10]). Processes of the form λ:I→I are called *numbers* or *scalars*.

For any system-type *A*, the identity process $id_{A}:A\to A$ represents “doing nothing” to any given “instance” of that system-type. The interaction of ∘, ⊗, and identity processes is subject to axioms guaranteeing that those two operations and the identity processes appropriately model sequential and parallel composition of processes. A precise definition of process theory in terms of *symmetric monoidal categories* follows. Further details on symmetric monoidal categories can be found in Mac Lane [11]. The process-theoretic language and diagrams in this paper largely follow the conventions in Coecke and Kissinger [12].

**Definition** **1.**  *A strict monoidal category* (C,⊗,I) *is a category* C *with a functor* ⊗:C×C→C *, called the monoidal product, and an object* I∈C *, called the monoidal unit, such that for objects* A,B, *and C and morphisms* f,g, *and h,*

- (A⊗B)⊗C=A⊗(B⊗C)
- (f⊗g)⊗h=f⊗(g⊗h)
- A⊗I=I⊗A=A
- $f⊗id_{I}=f=id_{I}⊗f$

*with* $id_{X}$ *denoting the identity morphism for any object X.*

Because ⊗ is a functor, if $f,f^{\prime },g,$ and $g^{\prime }$ are morphisms such that the composites $f^{\prime }\circ f$ and $g^{\prime }\circ g$ are defined, then

$$\left(f^{\prime }⊗g^{\prime }\right)\circ \left(f⊗g\right)=\left(f^{\prime }\circ f\right)⊗\left(g^{\prime }\circ g\right).$$

In this paper, morphisms are interpreted as processes, objects are interpreted as the input and output systems (more precisely, types of systems) of those processes, and the ordinary composition ∘ and monoidal product ⊗ are interpreted as operations for composing processes in sequence and in parallel, respectively. Therefore, it is useful for each category to have special morphisms for interchanging the order of systems, as in a circuit model of computation, where the lines representing two registers in a diagram may need to be brought together so that a box representing a gate can be applied to them.

**Definition 2.**  *A strict symmetric monoidal category is a strict monoidal category with an isomorphism $\sigma_{A,B}:A⊗B\to B⊗A$ for every pair of objects A,B such that for any morphisms $f:A\to A^{\prime },g:B\to B^{\prime }$ and any object C,*

$$\begin{array}{c} \sigma_{B,A}\circ \sigma_{A,B}=id_{A⊗B}\\ \sigma_{A,I}=id_{A}\\ \left(g⊗f\right)\circ \sigma_{A,B}=\sigma_{A^{\prime },B^{\prime }}\circ \left(f⊗g\right)\\ \left(id_{B}⊗\sigma_{A,C}\right)\circ \left(\sigma_{A,B}⊗id_{C}\right)=\sigma_{A,B⊗C}. \end{array}$$

A *process theory* is a strict symmetric monoidal category. Referring to a strict symmetric monoidal category as a process theory connotes a conception of the morphisms in the category as input–output processes; accordingly, to aid thinking and communication, we introduce the additional special terminology of system-types, processes, states, effects, and numbers/scalars for the objects and morphisms in a certain process theory. The isomorphisms $\sigma_{A,B}$ are called *swap maps*.

In this paper, processes are denoted by *string diagrams*, with generic processes depicted as boxes and objects as wires in diagrams read from bottom to top.

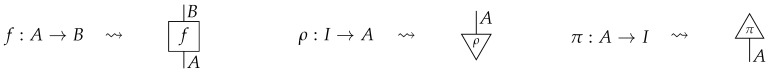


Boxes depicting states and effects may have either triangular or rectangular shape. Certain processes are depicted by special icons such as dots, with wires indicating their input and output system-types. The unit system-type *I* and identity process I→I are depicted by empty space. Other identity processes A→A are depicted by extensions of the wires that depict the objects.

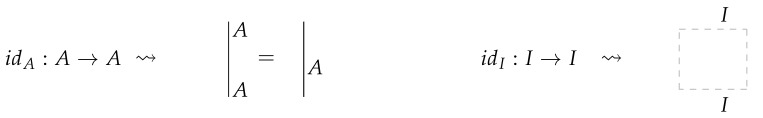


Sequential composition is indicated by boxes that are plugged together, while parallel composition is indicated by boxes or wires placed side-by-side (examples in the context of causal modeling can be found in Otsuka and Saigo [13]). Crossing wires indicate a swap map. The axioms for process theories ensure that a diagram deformed in such a way that connections are preserved represents the same process as the original.

### 2.2. The Process Theory $\mathrm{Mat}[R_{+}]$

All our causal models in this paper will involve a process theory of matrices of non-negative real numbers, within which processes on classical probabilistic systems with finitely many configurations can be modeled. This process theory, called $\mathrm{Mat}[R_{+}]$, has as system-types natural numbers, interpreted in this paper as the cardinalities of random variables. Sometimes, when discussing a generic system-type *A*, we want to refer specifically to the cardinality of the random variable associated with *A*; this cardinality is written |A|.

The processes M:m→n are the n×m matrices with entries that are non-negative real numbers $\left\{M_{j}^{i}\;|\;1\leq i\leq n,1\leq j\leq m\right\}$. Sequential composition is matrix multiplication; thus, identity maps are identity matrices.

Parallel composition of processes is given by the tensor product of matrices (aka the Kronecker product). The unit system-type is 1 (for which the identity process is the 1×1 matrix [1]:1→1); thus, scalars in this process theory are 1×1 matrices, or equivalently non-negative real numbers.

For each system-type *n* in $\mathrm{Mat}[R_{+}]$, let $d_{n}:n\to 1$ be the 1×n matrix (i.e., row vector) consisting of all 1s. This effect, called *discarding*, is depicted below.

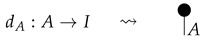


For a fixed system-type *A* in $\mathrm{Mat}[R_{+}]$, the state

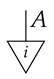

is the column vector with 1 in position *i* and 0s elsewhere, encoding certainty that a system is in the *i*th configuration. We sometimes call this state a “standard basis state”. The effect

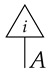

which we sometimes call a “standard basis effect”, is the matrix transpose of this state. For any labeled state (respectively, effect) in $\mathrm{Mat}[R_{+}]$, the effect (respectively, state) with the same label is provided by matrix transposition. We also call standard basis states *pure* states.

**Definition 3.**  *A process* f:A→B *in* $\mathrm{Mat}[R_{+}]$ *is called normalized if* $d_{B}\circ f=d_{A}$ *, or diagrammatically as shown below.*(3)
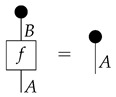

The normalization condition means that if the output of the process is discarded, then the actual process that took place is irrelevant. We can use Equation (3) to simplify composite processes by letting discard maps “fall through” normalized components.

From the definitions of discarding and normalization, it follows that normalized states in $\mathrm{Mat}[R_{+}]$ are column vectors with entries summing to 1 (i.e., probability distributions), while normalized processes are matrices with columns each summing to 1 (i.e., stochastic maps, equivalent to conditional probability distributions with $P\left(i|j\right):=M_{j}^{i}$).

For a normalized state ρ on system-type *A*, the composite 
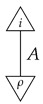

of ρ with effect *i* is the probability of a system described by a random variable with |A| values and distribution ρ being found in the *i*th configuration if checked. Effect *i* can be said to represent the property of being in the *i*th configuration.

The theory $\mathrm{Mat}[R_{+}]$ has a *copy* map for each system-type *A*, defined by(4)
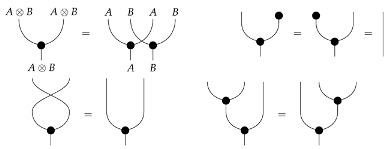

and the requirement that the copy map for *I* be $id_{I}$. In light of the last of the above equations, it makes sense to draw copy maps with arbitrarily many outputs, as shown below.

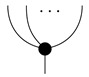

A copy map with one output is an identity map; a copy map with no outputs is a discard map. Process theories with such families of copy and discard maps [14] are called *copy-discard process theories*. Concretely, the copy map A→A⊗A is the matrix that copies pure normalized states; composing it with any of the |A| standard basis states i:I→A yields i⊗i.

### 2.3. Bayesian Networks in $\mathrm{Mat}[R_{+}]$

To understand how a graph such as the one in (1) constrains probability distributions (or, in causal inference, causal data-generating processes) but does not determine a single distribution (or data-generating process), it is useful to first associate with each graph a formal string diagram composed of labeled boxes and wires (and black dots), then consider the class of (usually many) processes in $\mathrm{Mat}[R_{+}]$ that can result from interpreting the boxes in the formal string diagram as specific matrices. The formal string diagram either depicts a process in a *syntactic* process theory defined by the graph [6], or is itself such a process [9]. We arrive at a specific distribution (or data-generating process) by interpreting the components of the formal diagram as specific normalized processes in $\mathrm{Mat}[R_{+}]$. In the causal context, we call the formal string diagram associated with a graph a *syntactic causal structure* and call its interpretations in $\mathrm{Mat}[R_{+}]$ *semantic causal structures*. For now, we will not distinguish carefully between a string diagram carrying a (perhaps arbitrary) interpretation in $\mathrm{Mat}[R_{+}]$ and one divorced from any interpretation; later, we introduce special notation for this distinction.

To form the abstract string diagram associated with a directed acyclic graph *G*, we first introduce a box $a:X_{1}⊗\ldots ⊗X_{n}\to A$ for every node *A* in *G* with parents $\left\{X_{1},\ldots ,X_{n}\right\}$. Each node in the graph now has an abstract *system-type label* that can appear on the input or output wires of boxes, and there is one box for each node. We compose these boxes by connecting each output *A* to an output of the overall diagram as well as to the inputs of each of the boxes associated with children of *A* in *G*, introducing copy maps where necessary. Calling a normalized state in $\mathrm{Mat}[R_{+}]$ Markov with respect to *G* means that it factorizes according to that diagram for some interpretation of the boxes as normalized processes (i.e., stochastic matrices). For example, a normalized state ω:I→A⊗B⊗C⊗D⊗E is Markov with respect to the graph *G* from (1) when it can be decomposed as shown below for some normalized processes a,b,c,d, and *e*.(5)
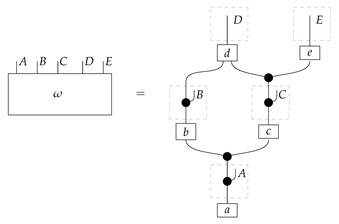

This diagrammatic condition corresponds precisely to the factorization of P(ABCDE) in (2), where P(ABCDE) is the joint probability distribution provided by the state ω. The diagram on the right-hand side of (5), with the boxes read as the appropriate stochastic matrices, is said to represent the state ω (i.e., the probability distribution P(ABCDE)) as a Bayesian network. Bayesian networks can efficiently represent probability distributions in many computational contexts. A Bayesian network representation allows a state on many variables (output system-types)–which generally requires a number of parameters exponential in the number of variables–to be specified by relatively few conditional probabilities. If each of five variables has cardinality 10, then a generic joint state on the five variables involves $10^{5}$ numbers. If the state is Markov-compatible with graph *G* in this example, it can be represented as a Bayesian network as in (5), and this representation involves only 1310 numbers: 10 for matrix *a*, 100 for each of the 10×10 matrices b,c, and *e*, and 1000 for the 10×100 matrix *d*.

Inference with Bayesian networks is efficient because updates can be made “locally” and then propagated only to those matrices where they might make a difference. In the current example, assume that a specific distribution Markov-compatible with *G* is stored as a Bayesian network. To update the distribution on A,B,D, and *E* using new knowledge that *C* has taken value *i*, we can compose the state ω with the effect i:C→I having 1 in position *i* and 0s elsewhere (the *i*th standard basis effect for *C*). Any standard basis effect *i* in $\mathrm{Mat}[R_{+}]$ satisfies(6)
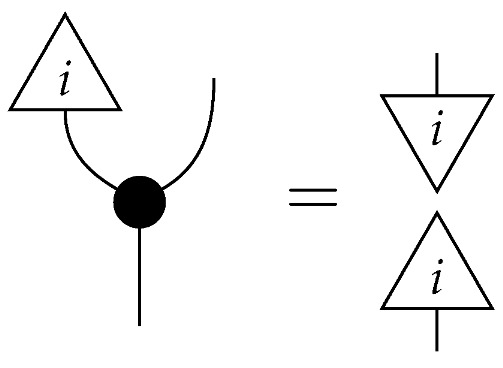

and the result is the same regardless of which of the two outputs of the copy map is plugged into the effect. Therefore, the updated state up to normalization is as follows.

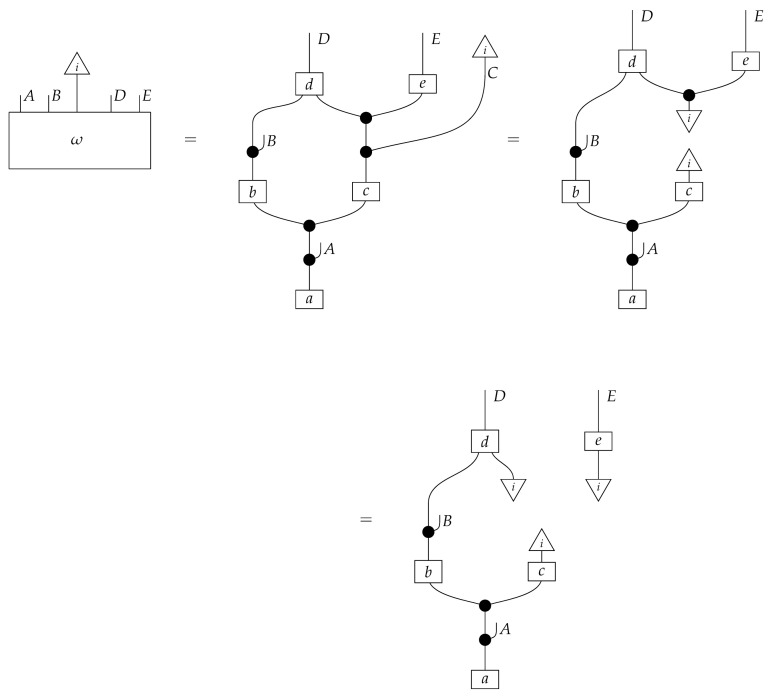

Suppose that we wish to know the new distribution for *E* alone. Up to normalization, this state is as follows.

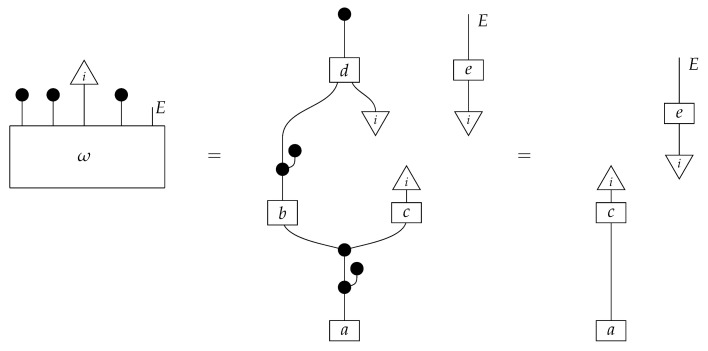

We can see that the normalized state will be some scalar times

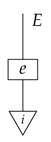

but the latter diagram is already a normalized state (it is a column of the matrix *e*). Therefore, we can obtain the new distribution for *E* simply by extracting the appropriate column of *e*. If we are only interested in this distribution, then we do not need to update any other part of the joint state.

### 2.4. Interventions and Causal Identification

For the kind of probabilistic inference just demonstrated, it does not matter why the joint distribution is known to be Markov-compatible with a particular graph, or equivalently, why the state is known to decompose according to a particular string diagram. In causal inference, on the other hand, it is assumed that the reason the state decomposes according to the string diagram is that the data have been generated by actual mechanisms represented by maps such as a,…,e. For each of these maps, the inputs and outputs are assumed to depend on those of the other maps in a manner corresponding to the connections in the diagram. Each box is understood to determine (stochastically) the value of its output variable given any value of its input variable, and to do so independently of the operation of the other mechanisms in the network. A concept of *local intervention* is introduced, with which we can imagine overriding the value of the variable associated with a single wire while leaving the rest of the network unchanged. In the running example, an intervention whereby variable *B* is forcibly set to a chosen value *i* is represented as follows.(7)
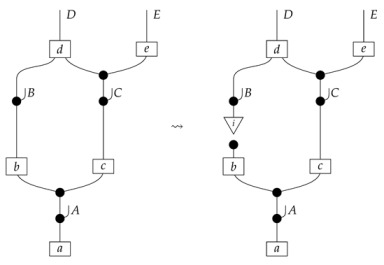

The output of process *b* is no longer input to *d*; instead, the state *i* is input. The intervention, which in Pearl’s formalism is implemented by the famous “do” operator, results in a different joint state from ω, called an *interventional distribution*. A Bayesian network understood as responding to interventions in this way is called a *causal Bayesian network*. A *causal identification* problem in the setting of causal Bayesian networks concerns inferring properties of the interventional distribution that a fixed, but initially incompletely known, causal Bayesian network would generate in response to certain hypothetical interventions. Interventions in a causal Bayesian network represent actions that for modeling purposes are considered performable at least “in principle” (where the meaning of “in principle” can vary with the application) in real-world causal scenarios associated with the network.

The inputs for an identification problem involving a causal Bayesian network include assumptions about the “shape” of the network and some of the data generated by the network. Typically, “shape” assumptions are simply given as the directed acyclic graph itself, with nodes labeled by variables; on the other hand, the data are given as a joint state resulting from no intervention, and as such are understood to represent an “observed distribution”. If the joint state has an output for every node of the graph, then the causal Bayesian network is called *Markovian*. On the other hand, the joint state may only have outputs corresponding to some but not all of the variables; in other words, some variables may be unobserved or “latent”, in which case the causal Bayesian network is called *non-Markovian*.

For example, one could be confronted with an unknown data-generating process satisfying causal assumptions given by the graph *G* in (1), with the observational distribution P(BCDE) of the form shown below.
(8)
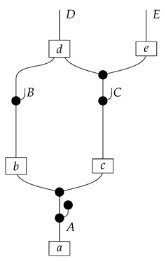


In this scenario, *A* is a latent variable; note that the extra copy of *A* output from the original state in (5) is discarded. One might be interested in the causal influence of *B* on *D*, meaning the distribution

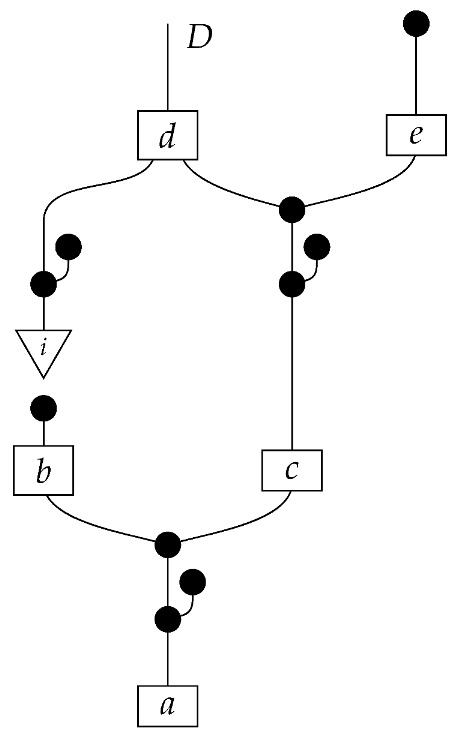

for every standard basis state *i* on *B*. In Pearl’s notation, such a distribution is written as P(D|do(B=i)). In general, the requested causal quantity may or may not be computable from the given information. There may be multiple causal Bayesian networks with the same graph that yield the same observational distribution (on a particular subset of the variables) but differ in the interventional distribution that one wants to infer. This is not the case for the current example, where despite the latent confounder *A* it is still possible to tell whether observed correlations between *B* and *D* are due to causal dependence of *D* on *B* or to joint dependence on variable *A*. However, if *C* were also latent, then distinguishing the causal influence of *B* on *D* from correlation underwritten by *A* would be impossible. Various criteria discovered over the years following Pearl’s introduction of the “do” operator (criteria summarized in Pearl’s book [1]) can indicate whether the causal quantity of interest is computable, or *identifiable*, from a graph and an observational distribution. These criteria have to do with what kinds of paths exist between those vertices in the graph labeled by observed variables and those labeled by latent variables.

Jacobs, Kissinger, and Zanasi [6] cast some of these graphical criteria (and the associated procedures for computing interventional distributions when they are identifiable) in terms of process theories. In their presentation, as in ours here, a graph specifies an abstract string diagram—a formal composition of labeled boxes and black dots—that encodes qualitative hypotheses for a causal scenario. That *syntactic causal structure* constrains numerical parameters such that the concrete data-generating process in any causal model based on that syntactic causal structure is consistent with the qualitative hypotheses encoded in the syntactic causal structure. Thus, the syntactic causal structure plays the same role in this framework as labeled graphs do in other presentations.The values of numerical parameters are specified by a *semantic causal structure*, in which each component of the syntactic causal structure is *interpreted* via a *functor of process theories* as a particular stochastic matrix in $\mathrm{Mat}[R_{+}]$. The entire semantic causal structure is a process in $\mathrm{Mat}[R_{+}]$ that determines (perhaps stochastically) the results of interventions in the causal scenario. Then, in an identification problem, one is given a graph—and consequently the syntactic causal structure—together with limited access to the semantic causal structure, typically in the form of an observational probability distribution. With these inputs, one tries to infer further properties of the semantic causal structure that permit the computation of interventional quantities. (Some authors use the term “causal structure” to refer only to the kinds of patterns of causal dependence typically specified by graphs; here, we use the word “structure” in a more mathematical sense. In general, many semantic causal structures will be consistent with a single syntactic causal structure, i.e., a single syntactic causal structure will have many interpretations. The goal of causal identification is roughly to figure out which of the possible semantic causal structures in fact characterizes a causal scenario assumed to be governed by a certain syntactic causal structure).

**Example 1.**  *It is well known that for any Markovian causal model based on a directed acyclic graph, any interventional quantity is identifiable from the graph and the probability distribution that the model generates under passive observation. Although this situation is sometimes described as “no intervention”, we de-emphasize this description, as later in this paper we treat passive observation as just one kind of intervention regime). In our terminology, we can state this result for the case of the graph G in* (1) *as follows.*

*When given as an input for causal identification, graph G tells us that the unknown causal model has the form on the right-hand side of* (5)*; that is, it tells us that that diagram is the scenario’s syntactic causal structure. Also given is the concrete probability distribution produced by the semantic causal structure. This distribution is encoded in the state ω, or equivalently in the following numbers:*

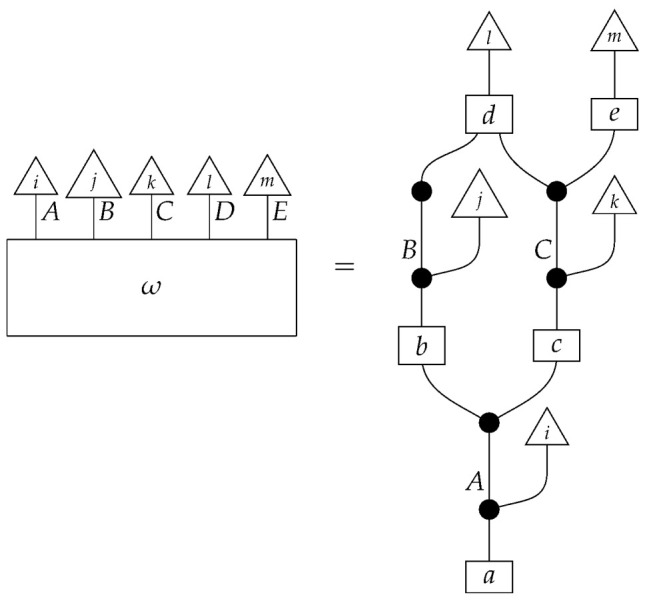

*where the effects i, j, k, l, and m run independently over the standard basis effects for the system-types A,B,C,D, and E in $\mathrm{Mat}[R_{+}]$ (there are |A| different effects i, |B| different effects j, etc.). The matrix values (i.e., interpretations) of the boxes are not given; knowing them would allow us to identify arbitrary interventional quantities of the kind exemplified in* (7)*.*

*As it happens, for any given normalized state (i.e., distribution) on A⊗B⊗C⊗D⊗E in $\mathrm{Mat}[R_{+}]$, there is only one joint interpretation of the boxes a,b,c,d, and e in* (5) *that gives rise to this particular state; that is, the values of the boxes (and consequently the response of the model to interventions) are determined by the overall state (observational data).*

According to Pearl [15], noting the identifiability of Markovian models is the beginning of causal analysis in graphical models. We will continue to concentrate on Markovian models, reformulating them to address the question of identifiability if the given probabilities result from noisy and disturbing observations.

## 3. Instruments, Tomography, Combs, and Examples of Causal Inference

Because a set of matrices of non-negative real numbers is closed under sums, every hom-set in $\mathrm{Mat}[R_{+}]$ can be given the structure of a commutative monoid. Moreover, this monoidal structure is compatible with both sequential and parallel composition of processes in the sense that (for instance) composing the zero matrix m→n (i.e., the additive identity element) in sequence or in parallel with another process yields another zero matrix, and addition is distributive over both sequential and parallel composition. This “enrichment” of the process theory in the category of commutative monoids (see Mac Lane [11] for further details) implies that sums distribute over entire diagrams. Sums can therefore be introduced without parenthetical grouping, as shown below.
(9)
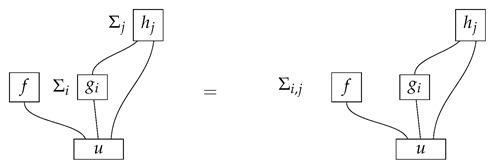


When composed with a normalized state, the sum of standard basis effects *i* and *j* provides the probability of the *i*th configuration plus the probability of the *j*th configuration. This sum can be said to represent the property of being in the *i*th or the *j*th configuration (for i≠j). The sum of all the effects *i* where the label *i* runs from 1 to |A| is the discarding map. This map’s composition with a normalized state yields the number 1. The sum can be said to represent the property of “being in some configuration”, which is satisfied with probability 1 by any system with a normalized state.

Sums in $\mathrm{Mat}[R_{+}]$ allow for the definition of mathematical entities representing quite generic ways in which agents may interact with processes. The concept of *instrument* used here is derived from quantum information science.

**Definition 4.**  *A $\mathrm{Mat}[R_{+}]$-valued instrument of type $A\to A^{\prime }$, where A and $A^{\prime }$ are system-types in $\mathrm{Mat}[R_{+}]$, is a finite set of maps, each of which has the form $\phi :A\to A^{\prime }$, and of which the sum is a normalized map. Each map ϕ is called a branch of the instrument.*

When an agent carries out a procedure represented by an instrument, one of the instrument’s branches is implemented and reported to the agent. The probability of a specific branch generally depends on both the branch and the process that the agent is probing with the instrument. An instrument with a single branch represents a procedure that results in the realization of that branch with probability 1.

**Example 2.**  *For system-type A in $\mathrm{Mat}[R_{+}]$, the |A| standard basis effects constitute an instrument of type A→I, as the sum of the standard basis effects is a discarding map, which is normalized. Suppose that an agent is confronted with (as many independent preparations as wanted of) an unknown normalized state ρ:I→A in $\mathrm{Mat}[R_{+}]$. When the agent “applies” the instrument to a single preparation, exactly one of the |A| effects/branches is realized; effect i is realized with probability i∘ρ. By applying the instrument to a large number of preparations of ρ, the agent can use the frequencies to estimate the probabilities i∘ρ. These probabilities determine the state ρ; indeed, they are the entries in the column vector.*

**Example 3.**  *Let $A^{\left(2\right)}$ denote the system-type of a binary random variable with values denoted* 1 *and* 2*. (Mathematically, the system-type is the number* 2*, corresponding to the number of values, but we adopt alternative notation to distinguish between the values and the system-type.) Let*

    ϕ=0.80.9    ϕ′=0.20.1.

*Effects ϕ and $\phi^{\prime }$ are the branches of an instrument of type $A^{\left(2\right)}\to I$. Suppose that we apply this instrument to an unknown normalized state $\rho :I\to A^{\left(2\right)}$) representing the variable’s distribution. On a single “trial”, if the variable’s true value is 1, then ϕ is realized with probability 0.8 and $\phi^{\prime }$ with probability 0.2. If the true value of the variable is 2, then ϕ is realized with probability 0.9 and $\phi^{\prime }$ with probability 0.1. On each trial, the received information is simply which of ϕ and $\phi^{\prime }$ has been realized. This information does not allow us to deduce from the trial which value the variable has taken, as we could in the previous example. Over a large number of trials, we can learn the probabilities ϕ∘ρ and $\phi^{\prime }\circ \rho$. Knowing the values of the instrument branches allows us to infer the entries in the vector ρ from these probabilities, i.e., to infer the unknown state.*

In the above example, branch ϕ has higher likelihood than branch $\phi^{\prime }$ for either value of the random variable modeled by ρ. However, the difference in relative likelihoods suffices for inference of ρ. A somewhat more intuitive example is one in which each value of the random variable makes a different instrument branch more likely but not certain, in such a way that the instrument represents a small deviation from the perfect observation in Example 2.

**Example 4.**  *Let*

    ϕ=0.80.1    ϕ′=0.20.9.

*If we consider ϕ and $\phi^{\prime }$ fuzzy versions of the predicate “has value* 1*” and “has value* 2*”, respectively, then the instrument with branches ϕ and $\phi^{\prime }$ “errs” twenty percent of the time when the true value of the random variable is 1 and ten percent of the time when true value is* 2*. If we know that we are using this instrument, we can infer an unknown state (on system-type $A^{\left(2\right)}$) exactly in the limit of infinitely many trials. Although this instrument is more informative on a single trial than the instrument in Example 3, the way in which the empirically revealed branch probabilities determine an initially unknown state is mathematically the same in both examples.*

**Example 5.**  *A discarding map $d_{A}$ in $\mathrm{Mat}[R_{+}]$ is the sole branch of an instrument of type A→I. An agent applying this instrument to an unknown normalized state on system-type A is told on each trial only that the single branch has been realized; the agent learns nothing about the state.*

**Example 6.**  *Let $A^{\left(3\right)}$ denote the system-type of a ternary random variable with values denoted* 1*,* 2*, and *3 *($A^{\left(3\right)}$ is convenient notation for the system-type* 3*). Let*

    ϕ=100.5    ϕ′=010.5.

*These two effects are the branches of an instrument of type $A^{\left(3\right)}\to I$. If we apply this instrument to an unknown normalized state on system-type $A^{\left(3\right)}$, then on a trial when we learn that ϕ has been realized, we can be certain that the true value of the random variable on that trial was not* 2*. If, on the other hand, we learn that $\phi^{\prime }$ has been realized, we can be certain that the true value of the random variable was not* 1*. This instrument, however, does not allow us to infer from infinitely many trials the value (i.e., probability distribution) of an arbitrary state. Let the arbitrary state be*

$$\left[\begin{array}{c} a\\ b\\ c \end{array}\right].$$

*The probabilities of ϕ and $\phi^{\prime }$ when the instrument is applied to this state are a+0.5c and b+0.5c, respectively. If we learn these probabilities from many trials, then we learn two linear equations in three unknowns. We also know the normalization condition a+b+c=1. These three equations do not generally have a unique solution for a,b, and c.*

*Now, suppose that we have access to both the instrument $\left\{\phi ,\phi^{\prime }\right\}$ and another instrument $\left\{\pi ,\pi^{\prime }\right\}$ with*

    π=001    π′=110.

*Although neither instrument by itself suffices to determine arbitrary unknown normalized states, both instruments together do suffice: with the entries in the state called a, b, and c, as above, using the first instrument allows us to estimate a+0.5c and b+0.5c, while using the second allows us to estimate c and a+b. The resulting system of linear equations in a,b, and c has a unique solution.*

The procedure of inferring an unknown state using the probabilities of various instrument branches (that may not be standard basis effects) is called *state tomography*. For an unknown state ρ and a known effect π of appropriate type, once the probability π∘ρ is known, it does not matter what instrument (of which π is a branch) was used to learn that probability. This irrelevance of the instrument under which an effect was realized extends to the instruments described below, for which the branches are not effects.

An agent’s preparation of a normalized state is represented by a one-branch instrument {ρ:I→A}. State preparation instruments can be used along with instruments made up of effects to perform *process tomography* on unknown normalized processes in $\mathrm{Mat}[R_{+}]$ with generic input and output system-types.

**Example 7.**  *Suppose that an agent is confronted with an unknown normalized process f:A→B in $\mathrm{Mat}[R_{+}]$. In addition, suppose that the agent has access to a |B|-branch instrument consisting of the standard basis effects for B and that for each standard basis state i:I→A the agent has access to state preparation instrument {ß:I→A} (that is, the agent can prepare any standard basis state at will). If the agent prepares standard basis state i at the input of f, then the result is the state shown below.*
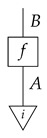
*If the agent now takes the instrument of type B → I consisting of the standard basis effects j and applies it at the output of f, then each branch j is realized with the following probability.*(10)
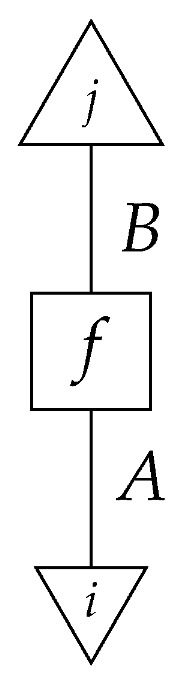
*The agent can learn these probabilities (for all standard basis effects j) by performing the experiment with the fixed state preparation instrument {i} for infinitely many trials. By repeating this procedure with the preparation instruments for all standard basis states i, the agent can learn the probabilities* (10) *for all i and j. These probabilities are the entries in the matrix f; therefore, the agent has now learned the value of f.*

As some of these examples of state tomography suggest, other instruments B→I could be substituted for the instrument consisting of the standard basis effects, and process tomography would remain possible. Process tomography would also remain possible if the standard basis state preparations were replaced by certain other state preparations.

**Example 8.**  *Let single-branch state preparation instruments {ψ} and $\left\{\psi^{\prime }\right\}$ be defined by*

    ψ=0.50.5    ψ′=0.90.1.

*These instruments together with the two instruments $\left\{\phi ,\phi^{\prime }\right\}$ and $\left\{\pi ,\pi^{\prime }\right\}$ of type $A^{\left(3\right)}\to I$ from Example 6 suffice for tomography of an unknown normalized process $f:A^{\left(2\right)}\to A^{\left(3\right)}$ in $\mathrm{Mat}[R_{+}]$. By applying each of the four possible combinations consisting of an instrument of type $I\to A^{\left(2\right)}$ and an instrument of type $A^{\left(3\right)}\to I$, we can learn the following probabilities from infinitely many trials:*

$$\begin{array}{ccccccc} & & & & \phi \circ f\circ \psi \; & \;\phi \circ f\circ \psi^{\prime } & \\ & & & & \phi^{\prime }\circ f\circ \psi \; & \;\phi^{\prime }\circ f\circ \psi^{\prime } & \\ & & & & \pi \circ f\circ \psi \; & \;\pi \circ f\circ \psi^{\prime }\\ & & & & \pi^{\prime }\circ f\circ \psi \; & \;\pi^{\prime }\circ f\circ \psi^{\prime }. \end{array}$$

*These numbers are not themselves the entries of the matrix f, but they allow the entries to be determined. Here, we have essentially performed a controlled experiment on the black-box process f, but it is controlled in an unusual sense, as we could not choose the value to feed into f, only which of two known distributions the value would be drawn from.*

What matters for the tomography of a process f:A→B in $\mathrm{Mat}[R_{+}]$ is whether we can prepare enough linearly independent states and whether enough linearly independent effects appear in the instruments of type B→I.

**Definition 5.**  *A set of effects* E={π:A→I} *in a process theory is called informationally complete for system-type A if any state* ρ:I→A *is uniquely determined by the set of numbers* {π∘ρ|π∈E} *. Similarly, a set of states* S={ρ:I→A} *is called informationally complete for A if any effect* π:A→I *is uniquely determined by the set of numbers* {π∘ρ|π∈S} *.*

**Example 9.**  *In $\mathrm{Mat}[R_{+}]$, an informationally complete set of states for system-type A is a set of |A| linearly independent column vectors of length |A|, while an informationally complete set of effects for A is the set of transposes of such a set of column vectors. In particular, the set of standard basis states on A is informationally complete, as is the associated set of effects.*

Tomography of arbitrary unknown normalized processes f:A→B (for fixed *A* and *B*) in $\mathrm{Mat}[R_{+}]$ is possible if one has access to preparation instruments for all of an informationally complete set of normalized states on *A* and access to instruments of type B→I for which union (a set of effects) is informationally complete for *B*.

The following property of $\mathrm{Mat}[R_{+}]$ regulates the tomography of processes with multiple inputs or multiple outputs.

**Proposition 1.**  *In the process theory $\mathrm{Mat}[R_{+}]$, any process*
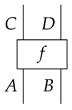
*is determined by numbers*(11)
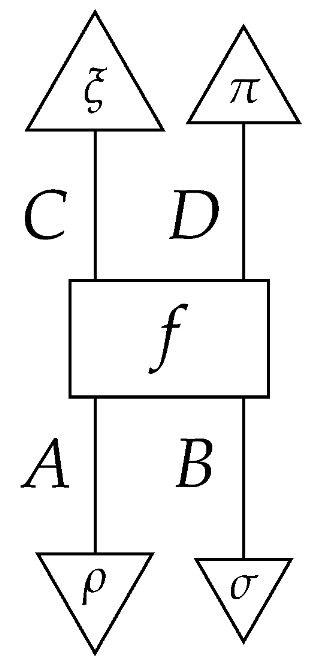
*where* ρ,σ,ξ, *and π index any informationally complete sets of states or effects for the appropriate system-types. We call this property tomographic locality.*

**Proof.**  The proposition follows from the prior characterization of informationally complete sets together with facts about tensor products in algebra, specifically, that tensor products of basis elements in two vector spaces span the tensor product of the spaces. □

Thus, one way to perform process tomography on an unknown normalized process f:A⊗B→C⊗D in $\mathrm{Mat}[R_{+}]$ is to select an informationally complete set of normalized states {ρ:I→A}, an informationally complete set of normalized states {σ:I→B}, an informationally complete set of effects {ξ:C→I}, and an informationally complete set of effects {π:D→I}, then learn the probabilities in (11). In terms of instruments, for every pair of states ρ and σ, it is necessary to probe *f* over many “trials”. On each trial, prepare the states ρ and σ on *f*’s inputs by applying the corresponding state preparation instruments, and measure *f*’s outputs with an instrument C→I and an instrument D→I for which the branches are the effects from {ξ:C→I} and {π:D→I}, respectively. (We assume for simplicity that each of the two sets of effects {ξ:C→I} and {π:D→I} forms a single instrument; in this case, only one pair of measurement instruments is needed for the process tomography protocol, while many single-branch state preparation instruments are needed. Alternatively, it may be that there are multiple instruments comprising different subsets of, e.g., {ξ:C→I}. Then, for each fixed pair ρ and σ, one would need to vary the measurement instrument C→I). On each trial with states ρ and σ, an effect ξ and an effect π will be realized. As the number of trials with fixed states ρ and σ approaches infinity, it becomes possible to estimate exactly the probabilities (11) for the fixed ρ and σ and for all ξ and π. By varying the states ρ and σ in this procedure, we can obtain all of the probabilities necessary to determine the value of *f*.

The causal models to be defined in this paper involve second-order processes that take various first-order processes as input and determine the probabilities of outcomes of either “observational” or more “interventional” interactions with systems. Thus, for example, a causal model based on the graph
(12)
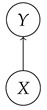

will involve a process
(13)
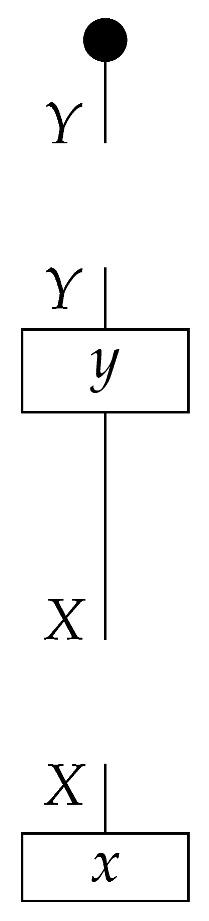

in $\mathrm{Mat}[R_{+}]$, where *x* and *y* are normalized. Each variable now corresponds to an input–output pair, depicted as a gap in the wire. This splitting of variables into an input and an output is similar to the situation in a single-world intervention graph [16], where nodes are split in two. The idea of a causal model as a second-order process with an input and an output for each node appears in works [17,18] focused on “quantum causal models”.

The gap formed by an input–output pair represents an *intervention locus* where a “system” arrives and then leaves, with an opportunity for interaction in between. The interactions in this case correspond to instruments of types X→X and Y→Y; that is, when an instrument is applied at a gap, a branch is realized and reported to the agent applying the instrument. The “state” of the system that is fed forward depends on which branch has been realized.

If f:X→X is a branch of the instrument applied at the *X* gap and g:Y→Y is a branch of the instrument applied at the *Y* gap, then the joint probability of realizing *f* and *g* is as follows.

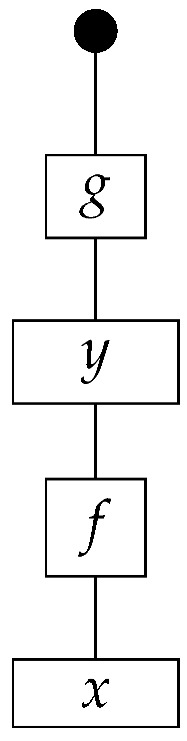

As shown below, the sum of these numbers over all pairs of branches *f* and *g* is 1, as the sums of the branches *f* and of the branches *g* are normalized maps. 
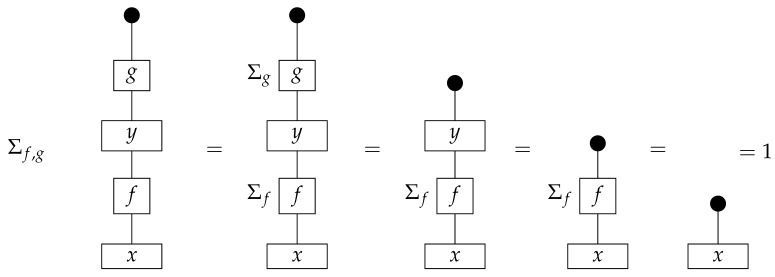
 Following the quantum information literature [19], we call second-order processes with gaps *combs*. Each of these combs can still be specified as an ordinary first-order process in $\mathrm{Mat}[R_{+}]$. Thus, the comb in (13) is a matrix X⊗Y→X⊗Y, and is equivalently depicted as follows.

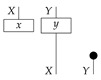

For technicalities regarding translation between the two representations—ensuring, e.g., consistency of calculations of the numbers generated by composition with processes X→X and Y→Y—see Jacobs, Kissinger, and Zanasi [6]. In this paper, all combs are depicted in the style of (13). It is useful to keep in mind, however, that a comb is ultimately a matrix of numbers (in our setting, a matrix of conditional probabilities governing a causal scenario); for our purposes, causal inference will involve learning these matrix entries by applying instruments.

**Example 10.**  *For any system-type A in $\mathrm{Mat}[R_{+}]$, there is an instrument A→A for which the only branch is $id_{A}$. Applying this instrument at a gap does not extract any information for the agent, and leaves the variable to pass through undisturbed.*

**Example 11.**  *For system-type A in $\mathrm{Mat}[R_{+}]$, the perfect passive observation instrument of type A→A has |A| branches forming a set (mathematically, the instrument is the set).*


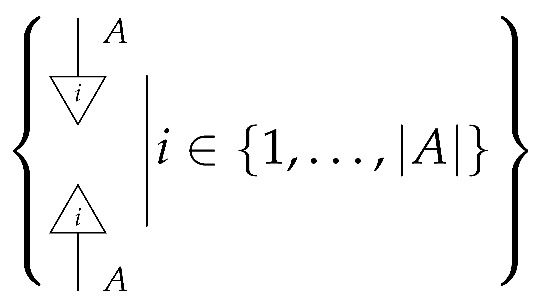

Knowing which branch of a perfect passive observation instrument has been realized means being certain of the value that a random variable has taken and being certain that the variable retains that value after observation.

**Example 12.**  *For the comb in* (13)*, for perfect passive observation instruments X→X and Y→Y at the X and Y loci, respectively, the joint branch probabilities are*
(14)
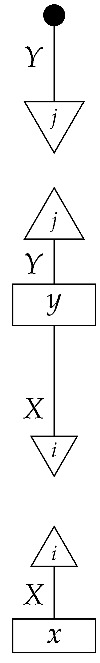

*where i runs from* 1 *to |X| and j from* 1 *to |Y|. If we apply the instruments for infinitely many trials and record the frequency of each pair of branches i∘i:X→X and j∘j:Y→Y, we learn a table indexed by i and j in which the entries are the probabilities in* (14)*. These probabilities would typically be written as P(X=i,Y=j). They factorize as expected for a causal model conforming to the graph* (12)*—they have the form P(X=i)P(Y=j|do(X=i)), or equivalently the form shown below.*

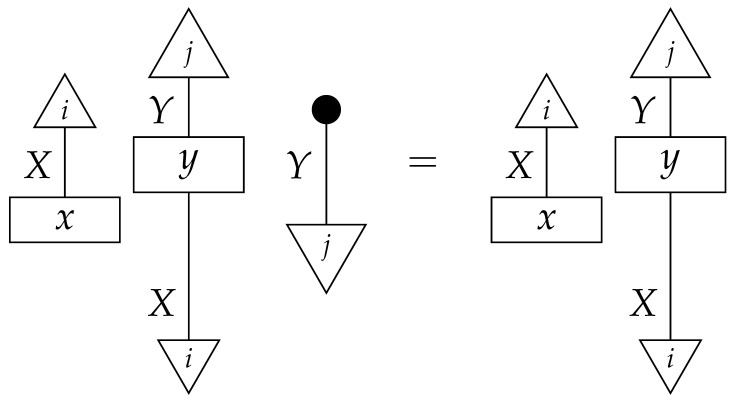

**Example 13.**  *Another important example of an instrument of type A→A that can be applied at a gap in a comb is one that discards the incoming system and prepares a fixed normalized state ψ for the outgoing system. A single-branch “discard-and-prepare” instrument has the following branch.*


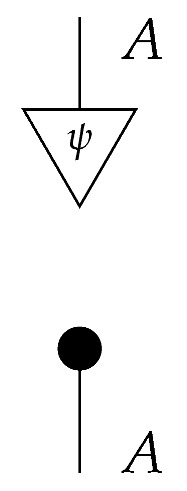


*If ψ is a standard basis state, then this instrument corresponds to what is called an “atomic” or “do” intervention. The state ψ may also be mixed, either because the agent carrying out the intervention procedure cannot fully control which value the variable has after the intervention—and the resulting uncertainty is represented by a specific probability distribution—or because the intervention procedure is designed to randomize the value in a specific way.*

*One example of a causal identification task is to use joint branch probabilities for perfect passive observation instruments in order to infer how perfect passive observations at one locus would turn out if a discard-and-prepare instrument were applied at another locus. Suppose that in the comb* (13)*, |X|=|Y|=2 and, initially unbeknownst to us, we have*

    x=0.80.2    y=0.60.30.40.7.

*We learn the probabilities* (14) *for all four pairs of values of i and j.*


j

12i10.480.32
20.060.14

*Now, we would like to infer the probability*(15)
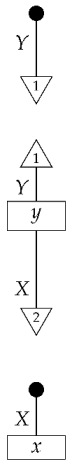
*i.e., the probability that passive observation at locus Y results in branch* 1 *if the value leaving X is forcibly set to* 2*. Because x and the standard basis state 1:I→Y are normalized, we know that among the three scalars in* (15)*, the top and bottom are each equal to* 1 *and the desired probability is as follows.*
(16)
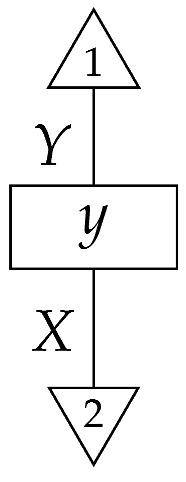

*The reader, knowing the value of the process y, can see that the desired probability is 0.3; however, from the perspective of the agents confronted with the inference problem, we do not yet know the value of y. We can transform the expression for the desired quantity into an expression with a value that is immediately computable from the known numbers* (14)*. First, we note the following:*

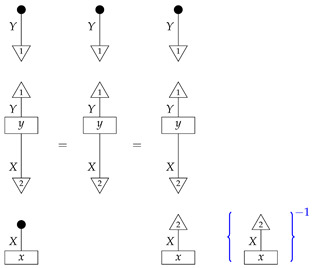

*where the inverse of a scalar is indicated by the scalar inside ${\{-\}}^{-1}$. Next, we rewrite the scalar being inverted in terms of numbers* (14).

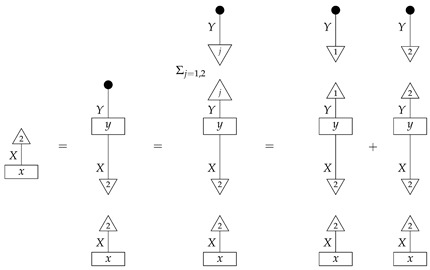

*For the first equality, we have used normalization of both y and a standard basis state; for the second equality, we have used normalization of the sum of the perfect passive observation branches at Y. The equation combining the far left-hand number with the far right-hand number essentially recovers the former from a joint probability distribution by marginalization.*

*We now know that the desired interventional probability is as follows:*


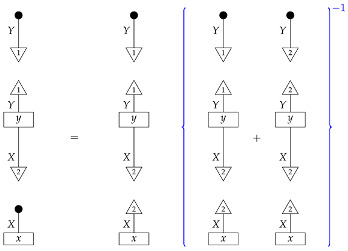


*which we can compute by referring to the table of joint probabilities from perfect passive observations; we obtain $0.06\times {\left(0.06+0.14\right)}^{-1}=0.3$, as the reader expected.*

The last example demonstrates how probabilities from perfect passive observation instruments are in fact what would normally be called observational probabilities. In our example, the “Markov assumption”, i.e., the assumption encoded in the graph (12) that the unknown comb has the form in (13), is used in the inference in the sense that the inference procedure does not apply to a two-locus comb of totally unknown shape. Of course, the direction of the edge indicates that the comb has locus *X* before locus *Y*; just as importantly, though, the Markov assumption rules out (most) combs of the form(17)
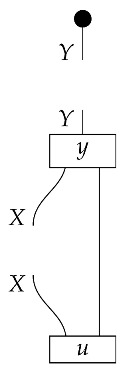

for which the just-presented inference protocol would not work. Put another way, for generic combs of the form in (17), the probabilities for perfect passive observation instruments at the *X* and *Y* loci do not uniquely determine the probabilities for discard-and-prepare instruments at the *X* locus and perfect passive observation instruments at the *Y* locus. This is essentially a statement of the well-known fact that observational data do not suffice to distinguish between causal influence of variable *X* on variable *Y* and joint influence of a latent confounder on both *X* and *Y*. To continue emphasizing the language of instruments, let us state explicitly what would suffice to determine the interventional quantity

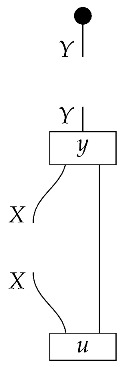

namely, the ability to implement the discard-and-prepare instrument with sole branch

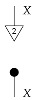

and implement a perfect passive observation instrument at the *Y* locus, for infinitely many trials.

**Example 14.**  *Let the effects ϕ and $\phi^{\prime }$ on system-type $A^{\left(2\right)}$ be as in Example 4:*

    ϕ=0.80.1    ϕ′=0.20.9.

*In addition, let*

    ψ=10    ψ′=01.

*Consider the instrument of type $A^{\left(2\right)}\to A^{\left(2\right)}$ of which the branches are ψ∘ϕ and $\psi^{\prime }\circ \phi^{\prime }$. Suppose that an agent applies this instrument to an intervention locus representing a binary variable. On a single trial, if the true value of the variable arriving at the locus is* 0*, then with probability 0.8, branch ψ∘ϕ is realized and reported to the agent, and value* 0 *is fed forward; with probability 0.2, branch $\psi^{\prime }\circ \phi^{\prime }$ is realized and reported and value 1 is fed forward. If the true value of the variable arriving at the locus is 1, then with probability 0.9 branch $\psi^{\prime }\circ \phi^{\prime }$ is realized and reported and value 1 is fed forward; with probability 0.1 branch ψ∘ϕ is realized and reported and value* 0 *fed forward. (The specification of the value that is fed forward in each case is redundant, as it follows from whether the branch that is realized on the trial contains state ψ or state $\psi^{\prime }$.) The agent knows the values of the branches, and for its own record-keeping can name branch ψ∘ϕ “*0*” and branch $\psi^{\prime }\circ \phi^{\prime }$ “*1*”. Now, on each trial, the instrument reports the correct value of the variable with probability 0.8 if the true value is 0 and with probability 0.9 if the true value is* 1*. Whichever value is reported is also fed forward; thus, when the instrument misreports the value of the variable, it also changes the value to match the report.*

*Now, suppose that we (taking the place of “the agent”) apply this instrument at the X locus in the comb* (13) *and a perfect passive observation instrument of type $A^{\left(2\right)}\to A^{\left(2\right)}$ at the Y locus. Again, |X|=|Y|=2. ($A^{\left(2\right)}$, X, and Y all denote the same system-type in $\mathrm{Mat}[R_{+}]$, but X and Y also denote loci and the associated input and output wires.) The matrix value of the comb, determined by the values of x and y in Example 13, is initially unknown to us, but we do know the comb shape, perhaps from the graph* (12)*. We are going to infer the value of the entire comb, which will allow us to infer the results of any counterfactual combination of instruments at the two loci.*

*The probabilities learned from infinitely many trials are the following.*


Y

i = 1i = 2X

ψ∘ϕ

0.3960.264


$\psi^{\prime }\circ \phi^{\prime }$

0.1020.238

*First, we determine the scalars ϕ∘x and $\phi^{\prime }\circ x$ by rewriting them in terms of probabilities we can look up in the table:*


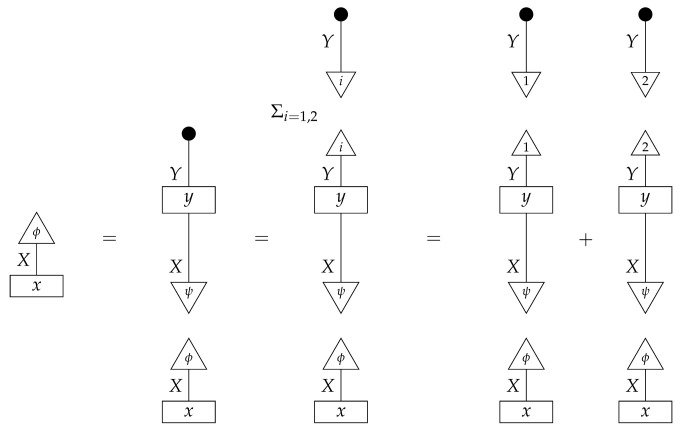


*and*


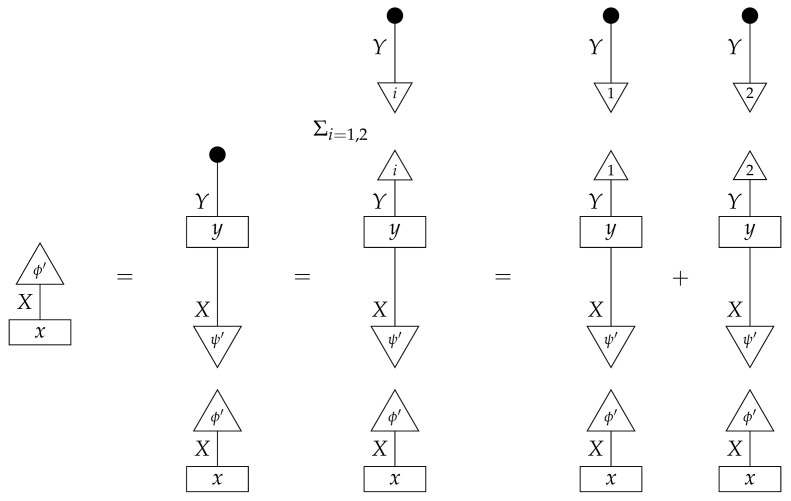


*thus, ϕ∘x=0.396+0.264=0.66 and $\phi^{\prime }\circ x=0.102+0.238=0.34$. These numbers allow us to determine the value of x, as the effects ϕ and $\phi^{\prime }$ form an informationally complete set. The computation of the matrix entries of x from these numbers is essentially a “change of basis” computation, and we omit it.*

*Now, we wish to determine the process y. Its matrix entries*


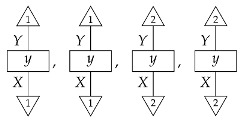


*are also known as*


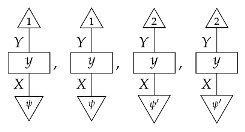


*respectively. We can compute each one using the already known numbers ϕ∘x and $\phi^{\prime }\circ x$ and the probabilities in the table. For example,*


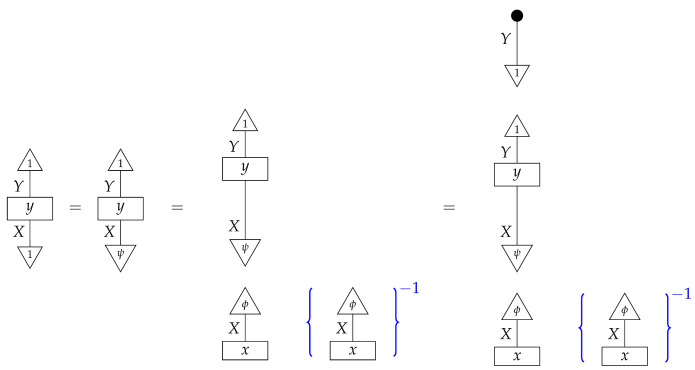


*that is, the (1,1) entry of y is the (ψ∘ϕ,1) entry of the table of branch probabilities times ${\left(\phi \circ x\right)}^{-1}$, or 0.396/0.66=0.6, as the reader expects from prior knowledge of the matrix y. We can compute the other entries of y similarly.*

In the next section, we are going to return to the language of syntactic and semantic causal structures to more rigorously reformulate the causal models from Section 2 (which in general include copy maps corresponding to forks in graphs) using combs and instruments, thereby treating the kind of observation implicit in the state-based representation on the same footing as other kinds of probing such as that represented by discard-and-prepare instruments or the instrument $\left\{\psi \circ \phi ,\psi^{\prime }\circ \phi^{\prime }\right\}$ in Example 14. The semantic causal structure involved in a causal model will be a comb in $\mathrm{Mat}[R_{+}]$ with an intervention locus for each variable, producing probabilities when instruments are assigned to all the loci. What are normally called “observational data” are then the probabilities generated under one particular instrument assignment, namely, the assignment of a perfect passive observation instrument to every locus.

For example, instead of the state in (5), a causal model based on the graph *G* in (1) can be represented with a comb A⊗B⊗C⊗D⊗E→A⊗B⊗C⊗D⊗E, as shown below.(18)
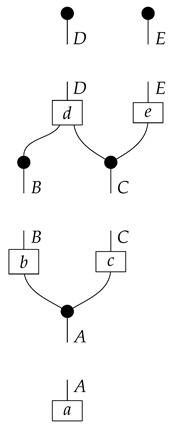

This comb (18) provides the observational data encoded in the state ω in (5): the probability of any particular joint outcome for the variables in joint state ω in (5) is provided by the composite of ω with the appropriate standard basis effects as shown below.
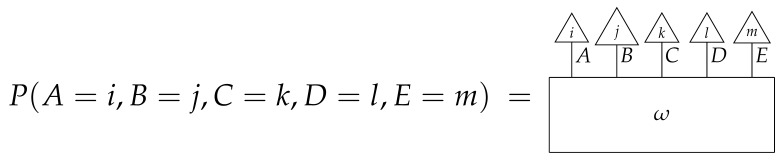

Per Equation (6), the right-hand expression is equal to the joint probability of perfect passive observation branches at all five gaps in the comb: (19)
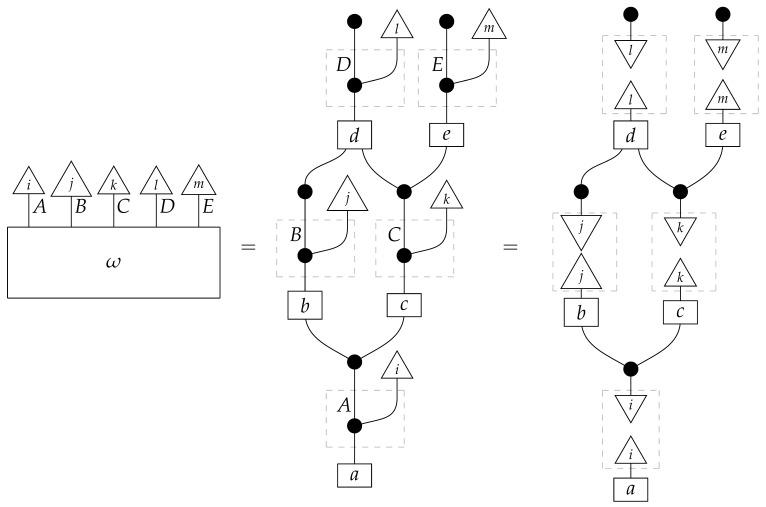

where the middle expression involves the original Bayesian network representation from (5). This equivalence helps to justify the definition of perfect passive observation instruments. Knowing the probabilities of “values of random variables” encoded in the state ω, i.e., knowing the state, is the same as knowing the outcome probabilities for perfect passive observation instruments.

The comb in (18) assigns probabilities to joint outcomes for any combination of instruments A→A, B→B, C→C, D→D, and E→E applied at the five gaps. For example, the joint probabilities for a discard-and-prepare instrument preparing standard basis state *i* at the *B* gap and perfect passive observation instruments at all other gaps follow.

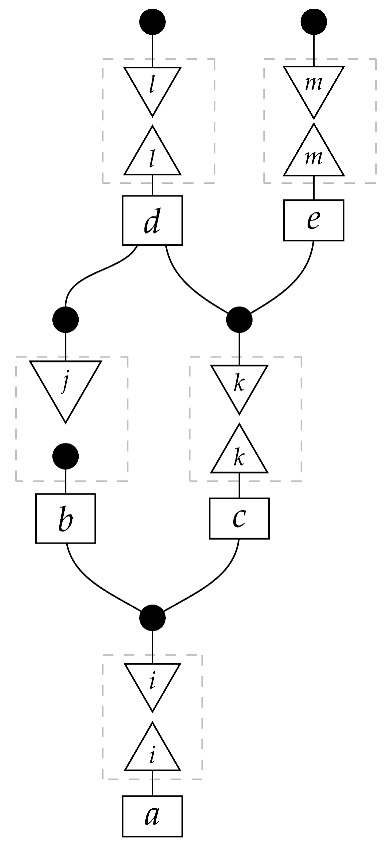


These probabilities are the same as those encoded in the joint state on the right-hand side of (7). In order to obtain the probabilities from (7), we simply compose the joint state with the appropriate standard basis effects. (There is a slight difference in the handling of discard-and-prepare interventions between representations such as (7) and the new comb representations. In the comb representation of the causal Bayesian network in our running example, if the single-branch discard-and-prepare instrument at the *B* gap prepares a mixed state ψ instead of a standard basis state *i*, then there is no way to model observation of the actual value about which ψ encodes uncertainty. In contrast, in the representation from (7) with *i* replaced by ψ, the copy map on *B* models perfect passive observation of variable *B* immediately after the preparation of ψ.)

By passing from states (as in (5)) to combs (as in (18)), we treat perfect passive observation as just one among many kinds of instrument that can in principle be applied to probe the process governing a causal scenario. Hence, we can precisely pose new kinds of causal identification problems such as the one solved in Example 14, by declaring that the statistics available for use in the inference are the outcome probabilities associated with instruments other than perfect passive observation. The task is then to use those statistics together with qualitative assumptions encoded in a graph to deduce a property of the data-generating process that implies how the process would respond to other instruments. For simplicity, we focus on identifying the value of the entire comb. In the next section, we will define in full the syntactic process theory associated with an arbitrary directed acyclic graph, explain how to construct the syntactic causal structure from the graph, and define causal models in terms of interpretation functors from the syntactic process theory into $\mathrm{Mat}[R_{+}]$.

## 4. Functorial Causal Models

Given a directed acyclic graph *G*, we formulate the syntactic causal structure for *G*-based Markovian causal models as a process in a syntactic process theory constructed as follows. First, we form a *free process theory*, i.e., a free strict symmetric monoidal category $\mathrm{Free}(\Sigma_{G})$ over a *signature* $\Sigma_{G}$ determined by the graph *G*. Here, a process-theoretic signature provides the symbols from which processes in the free process theory may be formed. It consists of a set of system-type symbols A,B,C,… and a set of labeled boxes and other icons, all of which have input and output wires labeled with system-type symbols. For each vertex *X* in *G*, the signature $\Sigma_{G}$ has a system-type symbol *X* and a box labeled *x* with an output wire labeled *X* and input wires labeled by the system-type symbols for *X*’s parents in *G*. Also for each vertex *X*, there are (i) an icon

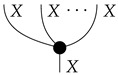

called “copy” with as many outputs as vertex *X* has children in *G*, and (ii) an icon

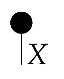

called “discard” or $d_{X}$. If vertex *X* has no children in *G*, then (i) and (ii) are identical.

The free process theory $\mathrm{Free}(\Sigma_{G})$ has the following system-types: a unit system-type *I*, the system-type symbols in $\Sigma_{G}$, and terms that are formal combinations of those symbols joined by ⊗, e.g., A⊗I⊗C⊗A⊗A⊗D. Its processes include identity maps for all system-types, swap maps for all pairs of system-types, and diagrams that can be formed from those maps and boxes in Σ via sequential and parallel composition. Processes are equal if and only if their diagrams are identical up to rearrangements that preserve connections.

To form the syntactic process theory $\mathrm{Syn}(\Sigma_{G})$ for *G*-based causal models, we impose equations between processes in $\mathrm{Free}(\Sigma_{G})$. “Imposing” equations means constructing a quotient process theory, which is defined as one wherein the system-types are the same as those of the original but wherein the processes are equivalence classes of processes from the original theory. We can treat individual representatives of the equivalence classes as being themselves processes in the quotient process theory, replacing them with other representatives at any point in a calculation. The equations defining $\mathrm{Syn}(\Sigma_{G})$ include those in (4), which make the “copy” icon behave as an abstract copy map that interacts with discarding in a way that mirrors the copy–discard interaction in $\mathrm{Mat}[R_{+}]$ ($\mathrm{Syn}(\Sigma_{G})$ is a copy–discard process theory), and equation

$$d_{B}\circ f=d_{A}$$

for every map f:A→B in $\mathrm{Free}(\Sigma_{G})$. The latter condition mirrors the normalization condition in $\mathrm{Mat}[R_{+}]$, and we accordingly call the processes in $\mathrm{Syn}(\Sigma_{G})$ (which all satisfy it) “normalized”.

The syntactic causal structure for graph *G* is now a process $c_{G}$ in $\mathrm{Syn}(\Sigma_{G})$, constructed as follows. For each vertex *X*, we connect the output wires of the *X* copy icon to the input wires of the boxes associated with *X*’s children. The resulting diagram has an input and an output wire for each vertex in *G*. We arrange these input–output pairs to form gaps in a comb.

For example, the graph in (1) gives as $c_{G}$ the process (18) (viewed as a formal diagram, not a matrix in $\mathrm{Mat}[R_{+}]$).

A semantic causal structure realizing the qualitative hypotheses in *G* is the image of $c_{G}$ under a certain *functor of process theories* $F:\mathrm{Syn}\left(\Sigma_{G}\right)\to \mathrm{Mat}\left[R_{+}\right]$, called an *interpretation functor*.

**Definition** **6.**  *For process theories* C and $C^{\prime }$ *with unit system-types I and* $I^{\prime }$ *and families of swap maps σ and* $\sigma^{\prime }$ *, respectively, a functor of process theories* $F:C\to C^{\prime }$ *is a strict symmetric monoidal functor, i.e., an ordinary functor of categories* $C\to C^{\prime }$ *, such that for system-types A and B in* C *:*

1. F(A⊗B)=F(A)⊗F(B)
2. $F\left(\sigma_{A,B}\right)=\sigma_{F(A),F(B)}^{\prime }$
3. $F\left(I\right)=I^{\prime }$ *.*

In order for a functor of process theories $F:\mathrm{Syn}\left(\Sigma_{G}\right)\to \mathrm{Mat}\left[R_{+}\right]$ to be a legitimate interpretation functor, we require that *F* send copy and discard maps in $\mathrm{Syn}(\Sigma_{G})$ to copy and discard maps in $\mathrm{Mat}[R_{+}]$, respectively (this requirement makes *F* a *copy–discard functor*). Normalization of all processes in $\mathrm{Syn}(\Sigma_{G})$ ensures that these processes’ images under *F* are stochastic matrices. In particular, the entire semantic causal structure $F(c_{G})$ is a stochastic matrix.

For a DAG *G*, a copy–discard functor $F:\mathrm{Syn}\left(\Sigma_{G}\right)\to \mathrm{Mat}\left[R_{+}\right]$ is called a *G-based causal model*. A *G*-based causal model implicitly includes the syntactic causal structure $c_{G}$ defined by *G* along with the semantic causal structure $F(c_{G})$. The same term will be used to refer to the semantic causal structure $F(c_{G})$.

### 4.1. Non-Markovian Models

A model constructed according to the procedure presented thus far is *Markovian*; each vertex in the graph has a corresponding input–output pair in the syntactic and semantic causal structures, meaning that the model mathematically allows us to consider probing at any node in the network. On the other hand, we may want to consider *non-Markovian* models based on a directed acyclic graph, in which some loci corresponding to vertices are “latent”. For a directed acyclic graph *G* with a list of vertices that are to correspond to latent loci, the prescription for forming the syntactic causal structure is as above, with one additional final step: for each locus that is to be latent, we join the output to the input with an identity map of the appropriate type, thereby closing the gap in the diagram.

In the example with graph (1), if *A* is to be the sole latent locus, then the syntactic causal structure is as follows.(20)
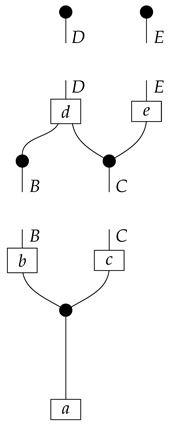


### 4.2. Causal Structures for ADMGs

More complicated kinds of graphs—featuring, e.g., bidirected edges or directed hyperedges—can represent causal hypotheses for non-Markovian scenarios without specifying all of the latent variables [20,21]. For example, the following Acyclic Directed Mixed Graph (ADMG)(21)
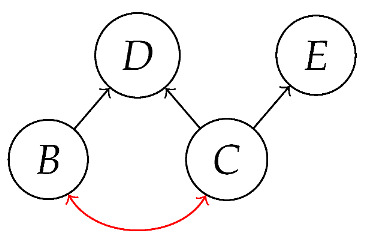

encodes, in addition to familiar hypotheses represented by black directed edges, the hypothesis (represented by a red bidirected edge) that there may be latent variables which simultaneously influence both *B* and *C*. Examples of DAGs consistent with these hypotheses are *G* from (1) with *A* latent,

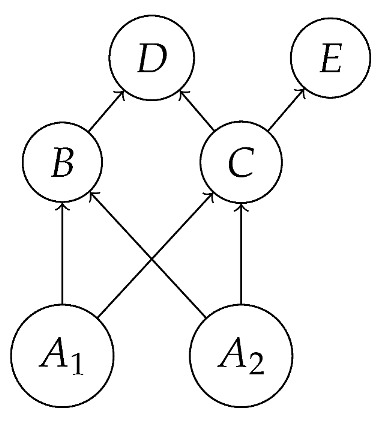

with $A_{1}$ and $A_{2}$ latent, and

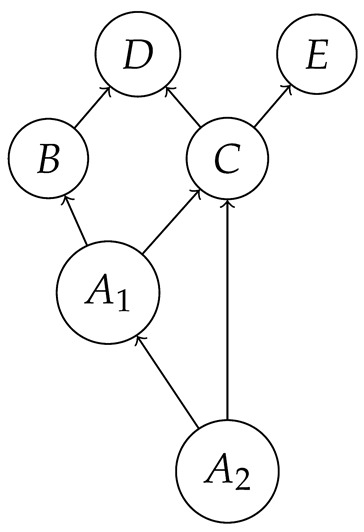

also with $A_{1}$ and $A_{2}$ latent.

It is possible to state a recipe for constructing a syntactic causal structure from an ADMG, similar to the way a syntactic causal structure is constructed from a DAG. The difference is that there are now processes (states) corresponding to bidirected edges. For example, the syntactic causal structure for the ADMG in (21) is as follows.(22)
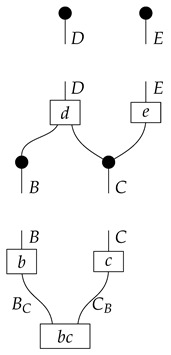


Again, a causal model based on the graph is provided by a copy–discard functor $F:\mathrm{Syn}\left(\Sigma_{G}\right)\to \mathrm{Mat}\left[R_{+}\right]$, and $F(c_{G})$ is called the semantic causal structure.

We leave a more thorough description of AMDG-based functorial causal models to other work; here, we simply note that the process-theoretic approach with both a syntactic and a semantic process theory allows us to understand very clearly how an ADMG represents “margins” of DAG-based models in the causal context. For example, at the level of syntactic causal structures, “marginalizing” (by plugging in an identity map) locus *A* in (18), which is based on the DAG in (1), yields (20), which also has the form (22) based on the ADMG (21). Alternatively, marginalizing in a single semantic interpretation of (18) results in a semantic causal structure that can be specified by a semantic interpretation of either (20) or (22).

## 5. Local Intervention Regimes and Identification

We use the term *intervention locus* to refer to an input–output pair in the semantic causal structure as well as to the corresponding input–output pair in the syntactic causal structure. If an instrument of type X→X is assigned to each locus *X*, then the semantic causal structure assigns probabilities to all possible combinations of branches. From here on, a single label will often stand for a locus (in both the syntactic and semantic causal structures), its input and output system-types in the syntactic causal structure, and its input and output system-types in the semantic causal structure.

**Definition 7.**  *A local intervention regime assigns an instrument of type* A→A *to each locus A in an interventional causal model.*

“Implementing” a local intervention regime for one iteration of a repeated causal scenario represented by a causal model results in the joint realization of a combination of maps at all the loci: at each locus, one branch of the instrument assigned to that locus is realized. The joint probability of this combination of local outcomes is the number resulting from plugging the maps into their loci in the semantic causal structure. The sense in which the causal models just defined are “causal” is that for a given local intervention regime, changing the instrument assigned to a locus (thereby creating a new local intervention regime that agrees with the first at all but one locus) might result in different outcome probabilities for the loci where the instruments are unchanged. In other words, a choice at one locus might “cause” events at other loci.

In a causal identification problem, one is given a graph (indicating a syntactic causal structure) and outcome probabilities from probing the initially unknown semantic causal structure with one or more local intervention regimes. Causal inference consists essentially in using outcome probabilities for some local intervention regimes to infer those for other local intervention regimes. The problem of causal identification is to use outcome probabilities from a limited set of local intervention regimes together with the shape of the syntactic causal structure (or the graph) to infer probabilities of outcome combinations under other local intervention regimes.

In the standard identification setup (e.g., in Pearl [1]), the probabilities available for inference are those that in our framework are generated under local intervention regimes consisting only of perfect passive observation instruments. We will study identification tasks based on data from other kinds of instruments.

The set of local intervention regimes whose outcome statistics are to be used for inference is specified as follows: for each locus *A*, an *accessible set* $I_{A}$ of instruments A→A is given. An *accessible* local intervention regime is then a local intervention regime in which each instrument comes from the accessible set for the locus to which the instrument is assigned. The probabilities available for inference are the probabilities that can be “learned” from accessible local intervention regimes—that is, for each accessible local intervention regime, the joint probability of each combination of branches will be considered known.

The quantity to be identified in a causal identification problem is the image of some map in the syntactic process theory under the interpretation functor *F*. In this paper, we focus for simplicity on identifying the entire semantic causal structure $F(c_{G})$.

In full, the causal identification problem studied in this paper is defined by the following in each instance: a causal model consisting of a directed acyclic graph *G*, a set of latent loci and an interpretation functor *F*, and an accessible set $I_{A}$ of instruments for each locus *A*. The inputs for the identification task are the graph *G* and the set of latent loci—which together prescribe how to construct the syntactic causal structure $c_{G}$—along with the data generated by $F(c_{G})$ under each accessible local intervention regime. For each accessible local intervention regime, a table is provided in which every combination of instrument branches realizable at the various loci on a single “trial” is associated with its probability. The collection of tables specifies the set of accessible local intervention regimes, and consequently the accessible sets of instruments. The probabilities in these tables are called *accessible probabilities*. The task is to compute $F(c_{G})$. If this task is possible, we say that $F(c_{G})$ (or “the model”) is *identifiable* from the accessible sets of instruments (and the graph with the list of latent loci).

We assume from here on that all DAG-based models are *strictly positive* in the following sense (it is straightforward to formulate the corresponding assumption for models based on ADMGs and other kinds of decorated graphs): a causal model *F* based on a DAG *G* is called strictly positive if, for every process *x* in $\mathrm{Syn}(\Sigma_{G})$ corresponding to a vertex *X* in *G*, the stochastic matrix F(x) in $\mathrm{Mat}[R_{+}]$ has only strictly positive entries. Our strict positivity assumption plays a similar role to that of requirements in the standard causal inference literature that distributions be strictly positive; in both cases, strict positivity guarantees that the conditional probabilities needed for calculations of interventional quantities are defined. Intuitively, if one wants to infer a “do”-conditional probability P(Y=y|do(X=i)) from observational data, one needs the possibility X=i to be realized in those data. Our restriction to strictly positive models brings the following fact into our calculations: in $\mathrm{Mat}[R_{+}]$, when a matrix *f* with only strictly positive entries is composed with a state ρ, then as long as some entries in ρ are non-zero, the resulting state f∘ρ has only strictly positive entries. If *f* and ρ are normalized, then f∘ρ corresponds to a strictly positive probability distribution.

Our version of the most common kind of identification task involves accessible sets consisting only of perfect passive observation instruments (each accessible set is a singleton). In these cases, we speak of quantities being identifiable “from perfect passive observation”.

**Proposition 2.**  *Models based on the DAG in* (1) *with A and C latent or based on the ADMG in* (21) *are not identifiable from perfect passive observation.*

**Proof.**  As in the discussion of comb shape (17), this is an example illustrating our framework using an established fact from the causal inference literature: in general, (interventional quantities associated with) non-Markovian models are not identifiable from “observational data”. □

**Proposition 3.**  *Models based on the DAG in* (1) *with only A being latent are identifiable from perfect passive observation.*

Put another way, any semantic interpretation of (20) is discernible from knowledge of the syntactic causal structure and the statistics from a local intervention regime consisting only of perfect passive observation instruments.

From here on, we distinguish boxes in a syntactic process theory from their images in $\mathrm{Mat}[R_{+}]$ by indicating the latter in boldface; thus, x indicates the image F(x) of a process *x* under interpretation functor *F*.

**Proof.**  An arbitrary semantic causal structure is

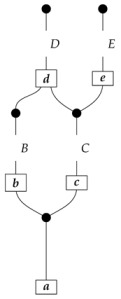

and the accessible probabilities are

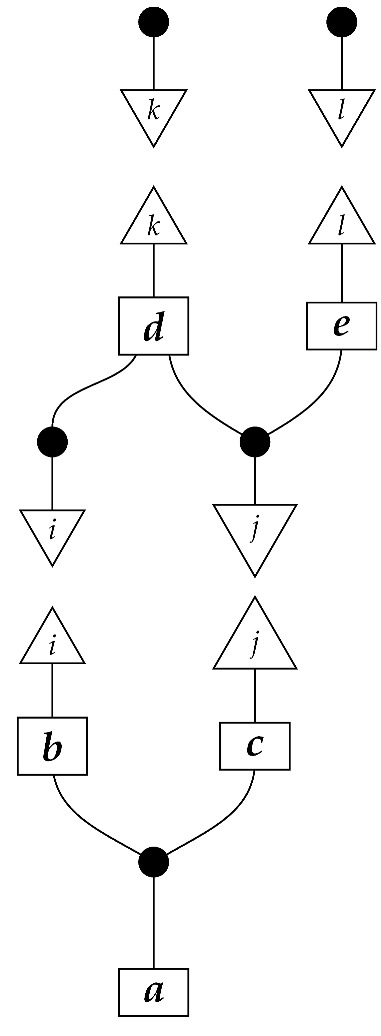

where the indices i,j,k, and *l* take values from 1 to |B|,|C|,|D|, and |E|, respectively. Identification proceeds in several steps. First, we learn the value of
(23)
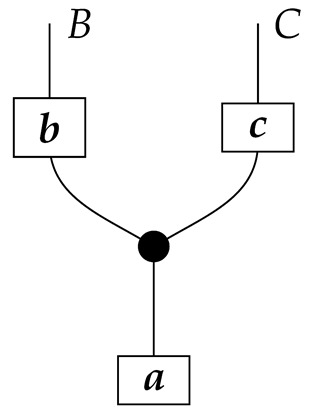

by computing its matrix entries

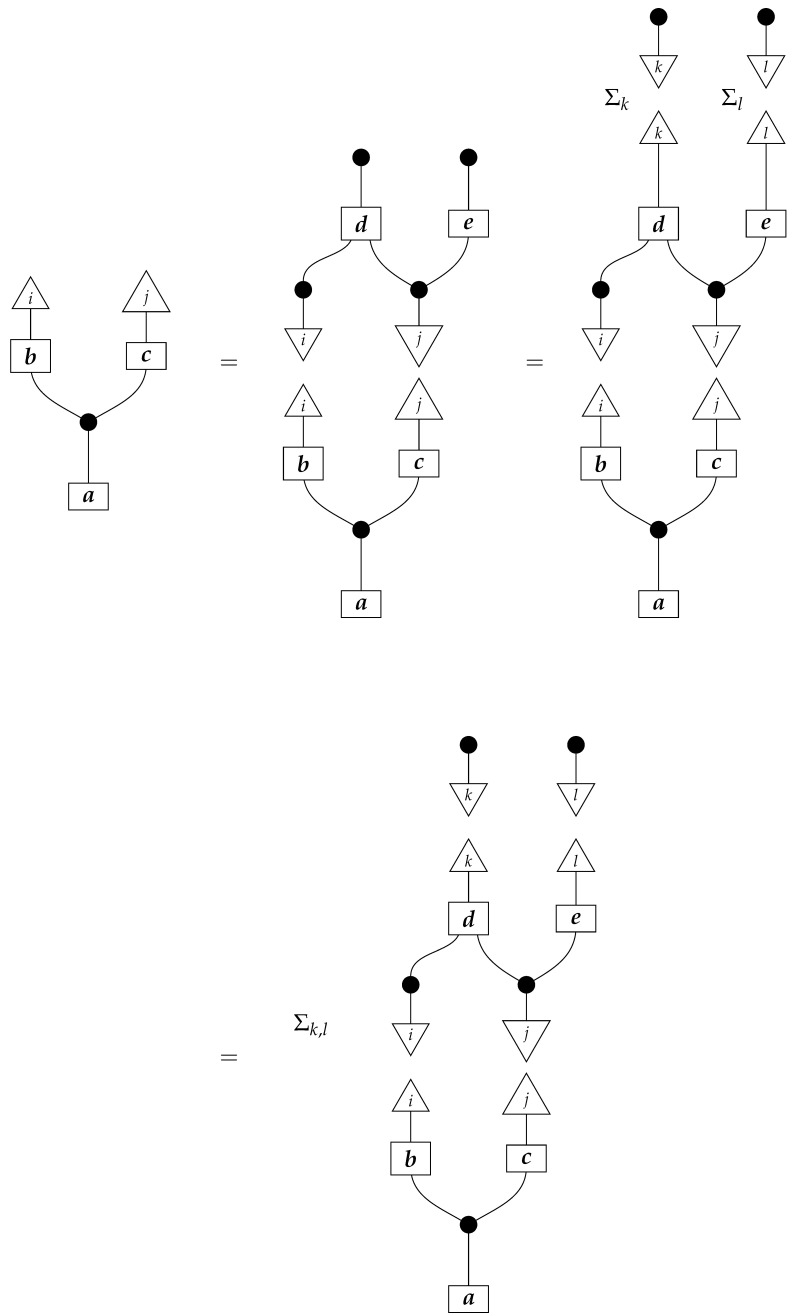

The reason for the first equality is that

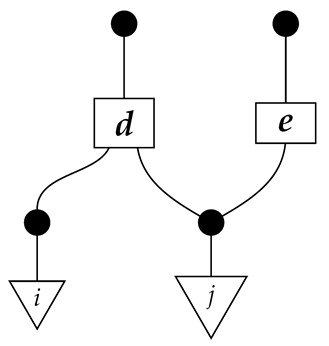

is the scalar 1, since d and e are normalized; the second equality follows from the fact that adding the branches of an instrument (here, a perfect passive observation instrument) results in a normalized map (here, an identity map), which discarding “falls through”.

Next, we use the matrix entries of (23) together with the accessible probabilities to compute the process(24)
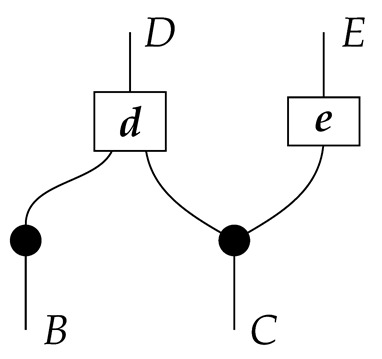

for which the matrix elements are

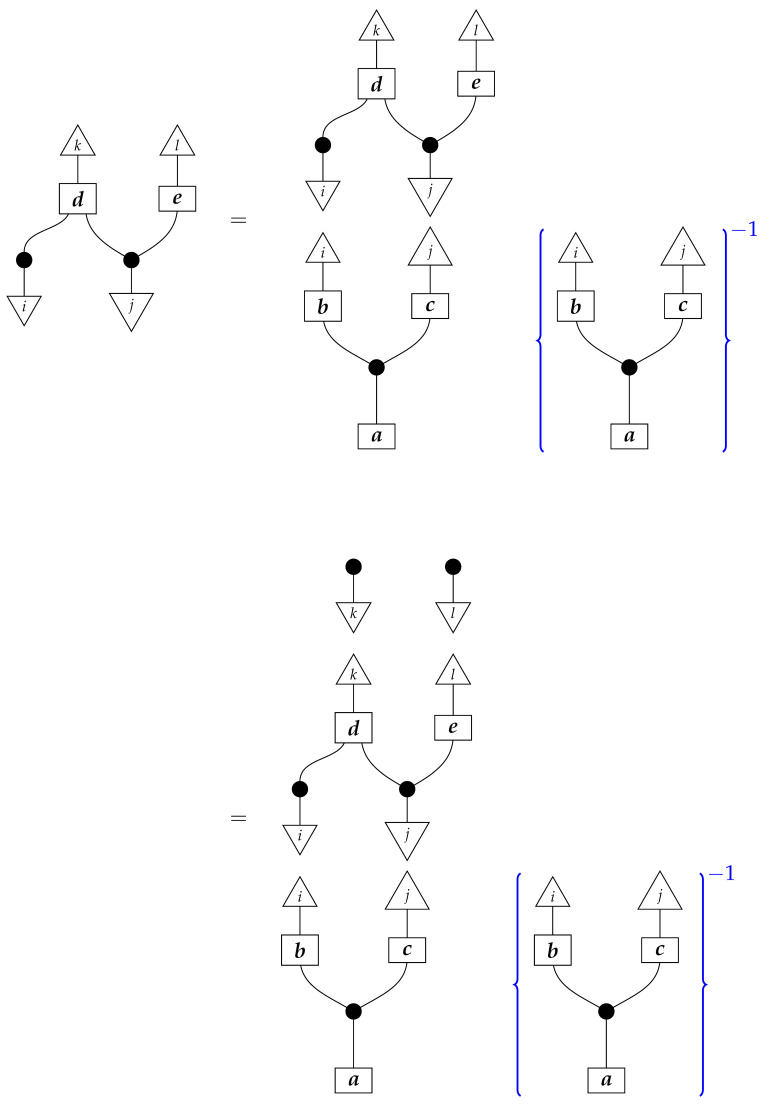

where the right-hand side is in the form of a product of an accessible probability and the inverse of a number already known from the previous step.

We now know the values of processes (23) and (24), which together with the two discard maps make up the semantic causal structure; hence, we know the semantic causal structure. □

This proof has two notable features. First, it was not necessary to compute the value of the image of every box in $\mathrm{Syn}(\Sigma_{G})$ on its own. Indeed, doing so is impossible in this example, as the state (23) (equivalently, its matrix entries) does not fix unique values of the matrices a,b, and c. Second, the accessible instruments were perfect passive observation instruments; thus, we computed matrix entries rather directly from accessible probabilities, in the sense that the accessible probabilities were products of matrix entries of processes in the semantic causal structure. This would not be the case for accessible instruments other than perfect passive observation instruments, but it might still be possible to compute the values of component processes in the semantic causal structure. For example, if we could somehow obtain the numbers

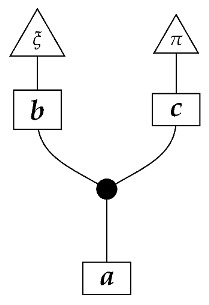

from the accessible probabilities for informationally complete sets of effects {ξ} and {σ}, then, even if the effects were not standard basis effects, we would be able to compute the value of the process in (23).

**Proposition 4.**  *Markovian models based on DAGs are identifiable from perfect passive observation.*

**Proof.**  This is a statement of a well-known fact represented in our framework. It follows from the uniqueness of factorization described in Example 1 together with the equivalence between the matrix entries of a state and the probabilities of perfect passive observation branches, as in (19). □

The proposition for Markovian models defined with combs in this paper follows from Theorem 1 below. For now, we can consider the representation of a DAG-based causal model as a state (for example, in (5)). In causal identification from perfect passive observation, the state (i.e., probability distribution) ω is provided together with the graph *G* indicating that the causal mechanisms a,b,c,d, and *e* are arranged as in (5). A special feature of a Markovian model—i.e., one in which each box’s output is copied to an output of the overall state, as opposed to being discarded as in (8)—is that the probability distribution is consistent with only one set of values for the five mechanisms. The five factors on the right-hand side of P(ABCDE)=P(A)P(B|A)P(C|A)P(D|BC)P(E|C) of the observational distribution specify the five stochastic matrices in (5). In other words, for a Markovian model, the observed conditional probabilities are also the values of the stable and autonomous mechanisms. This narrative for the state-based representation suggests that it is the Markovianity of a *probability distribution* relative to a DAG that allows for the identification of a Markovian *causal model* based on that DAG. We will show with the comb representation that Markovianity of accessible probability distributions need not be understood as the condition making Markovian models identifiable. If the accessible instruments satisfy a certain abstract condition (met in particular by perfect passive observation instruments and some other instruments in Section 3), then Markovian models are identifiable even though the accessible probability distributions may not be Markovian for the graphs.

**Definition** **8.**  *A set of maps* $\phi :A\to A^{\prime }$ *in* $\mathrm{Mat}[R_{+}]$ *is called marginally informationally complete if it satisfies both of the following conditions:*

*1.*  *The set of effects 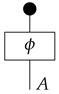 is informationally complete for A.*
*2.*  *For any normalized state* $\rho :I\to A^{\prime }$ *with full support, the set of states 
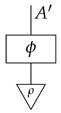
 is informationally complete for* $A^{\prime }$ *.*

The following proposition follows from Proposition 6 below.

**Proposition** **5.**  *The set of branches of a perfect passive observation instrument A→A is marginally informationally complete.*

**Definition 9.**  *A set of instruments of type $A\to A^{\prime }$ is called marginally informationally complete if the union of the instruments (with each instrument viewed as a set of maps) is a marginally informationally complete set of maps.*

Thus, Proposition 5 states that a perfect passive observation instrument by itself constitutes a marginally informationally complete set of instruments.

The following two examples include assertions of marginal informational completeness of certain singleton sets of instruments, justified by Proposition 6 below.

**Example 15.**  *Consider a locus A corresponding to a binary random variable (i.e., |A|=2). Suppose that one is able to probe it with an instrument that reports the true value of the variable arriving at that locus, but that also disturbs the variable such that the value to be fed forward is flipped with probability 0.1. The branches of this instrument are the following:*

$$\begin{array}{ccccccccccc} & & & & \left[\begin{array}{c} 0.9\\ 0.1 \end{array}\right]\circ \left[\begin{array}{cc} 1 & 0 \end{array}\right] & , & & & & & \left[\begin{array}{c} 0.1\\ 0.9 \end{array}\right]\circ \left[\begin{array}{cc} 0 & 1 \end{array}\right]. \end{array}$$

*The set containing only this instrument is marginally informationally complete.*

It is not necessary that the disturbance be “symmetric”, as in this instrument, nor that the effects be standard basis effects.

**Example 16.**  *Let*

    ϕ=0.80.9    ϕ′=0.20.1    ψ=0.50.5    ψ′=0.90.1.

*The instrument with branches ψ∘ϕ and $\psi^{\prime }\circ \phi^{\prime }$ constitutes a single-instrument marginally informationally complete set of instruments of type $A^{\left(2\right)}\to A^{\left(2\right)}$ (recall that we use the notation $A^{\left(2\right)}$ for the system-type* 2*, in order to reserve the notation “*2*” for a value of the random variable). When a locus representing a binary random variable (with values denoted* 1 *and* 2*) is probed with this instrument, if the variable’s true value is* 1*, then ψ∘ϕ is realized with probability 0.8 and $\psi^{\prime }\circ \phi^{\prime }$ with probability 0.2. If the true value of the variable is* 2*, then ψ∘ϕ is realized with probability 0.9 and $\psi^{\prime }\circ \phi^{\prime }$ with probability 0.1. If branch ψ∘ϕ is realized, then the value of the variable fed forward after the probing is totally randomized. If branch $\psi^{\prime }\circ \phi^{\prime }$ is realized, then the value fed forward is* 1 *with probability 0.9 and* 2 *with probability 0.1.*

The instruments in these examples have the property that each branch has the form ψ∘ϕ for an effect ϕ and a state ψ, i.e., each branch is *∘-separable*. If all instruments in a set consist only of ∘-separable branches, then marginal informational completeness of the set of instruments can be checked with the following criterion.

**Proposition** **6.**  *A set of maps* $\psi \circ \phi :A\to A^{\prime }$ *in* $\mathrm{Mat}[R_{+}]$ *composed of states* $\psi :I\to A^{\prime }$ *and effects* ϕ:A→I *is marginally informationally complete if and only if: (i) the set of all effects ϕ is informationally complete for A, and (ii) the set of all states ψ is informationally complete for* $A^{\prime }$ *.*

**Proof.**  Suppose that we have a set of maps
(25)
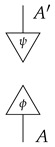

in $\mathrm{Mat}[R_{+}]$ such that the set of effects ϕ is informationally complete for *A* and the set of states ψ is informationally complete for $A^{\prime }$.

(i) The set of effects

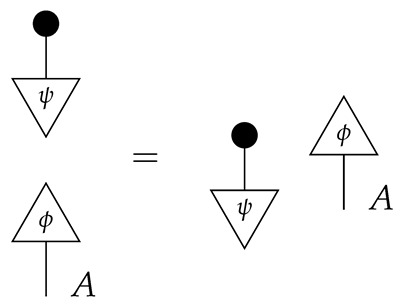

is informationally complete for *A*, as scaling each of a linearly independent set of row vectors results in a linearly independent set.
(ii) For any state $\rho :I\to A^{\prime }$ with full support, the set of states

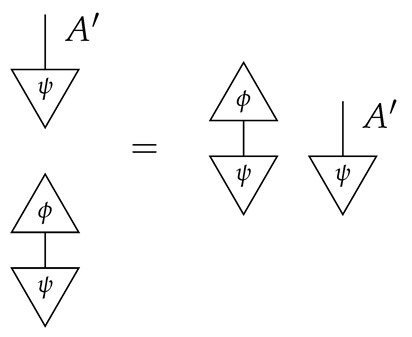

is informationally complete for $A^{\prime }$ by a similar consideration about preservation of linear independence under scaling.

Conversely, suppose that the set of maps (25) satisfies conditions 1 and 2 in Definition 8. Then, the effects

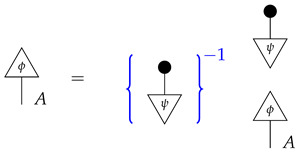

are re-scalings of the effects *d_A′_* ∘ *ψ* ∘ *ϕ* supposed to form an informationally complete set. To see that the states ψ form an informationally complete set, we can plug a fixed state $\rho :I\to A^{\prime }$ with full support into each of the maps (25). The resulting states ψ∘ϕ∘ρ on $A^{\prime }$ are supposed to form an informationally complete set, and the states

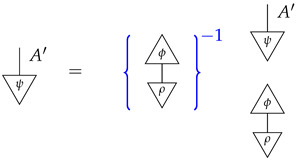

are re-scalings of these states. □

We would also like to deal with instruments for which the branches are not ∘-separable. For a given locus, consider a probing procedure for which one of the possible outcomes tells the observer the following: the variable may have had either value 1 or 2 when arriving at the locus, and both possibilities should be assigned equal probability, but with certainty that the value upon leaving the locus is the same as the value upon arriving. The instrument branch corresponding to this outcome is

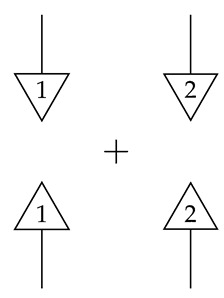

which is non-∘-separable. An instrument with branches that are sums of perfect passive observation branches represents an observation that is “imperfect” or “coarse-grained” but not “disturbing”. Loosely speaking, although a ∘-separable instrument can represent a procedure for which outcomes carry only incomplete “information” about the values arriving at and leaving the locus, it cannot represent a procedure that results in “shared information” between the incoming and outgoing systems that is inaccessible to the observer. However, procedures of the latter sort, such as coarse-grained non-disturbing observations, are among the first that come to mind when one thinks of generalizing from perfect passive observation. The (classical version of the) identifiability result demonstrated for a three-node graph in [9] covers only ∘-separable instruments. The main result of the present paper, in Theorem 1, is that a Markovian model is identifiable whenever the accessible set of instruments at each locus is marginally informationally complete regardless of whether or not all the accessible instruments are ∘-separable.

**Theorem 1.**  *Markovian models are identifiable whenever the accessible set of instruments at each locus is marginally informationally complete.*

We begin by demonstrating identifiability for the example of three-locus models based on the graph(26)
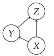

with the following syntactic causal structure.

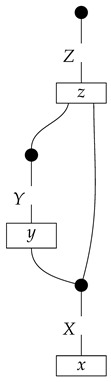


A semantic causal structure has the form shown below.

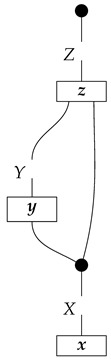


First, x is determined by the probabilities formed by its composition with the informationally complete set of effects $d_{X}\circ f$, with the maps *f* forming a marginally informationally complete set. These probabilities are obtained from accessible probabilities via marginalization of the outcomes at *Y* and *Z*. Here, *g* and *h* vary over the branches of single instruments; that is, all the local intervention regimes from which data are collated share the same choices of instruments at *Y* and *Z*.

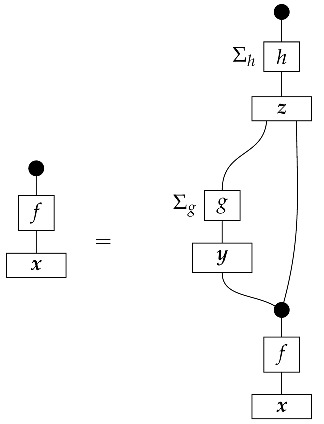


Next, y is determined as follows:

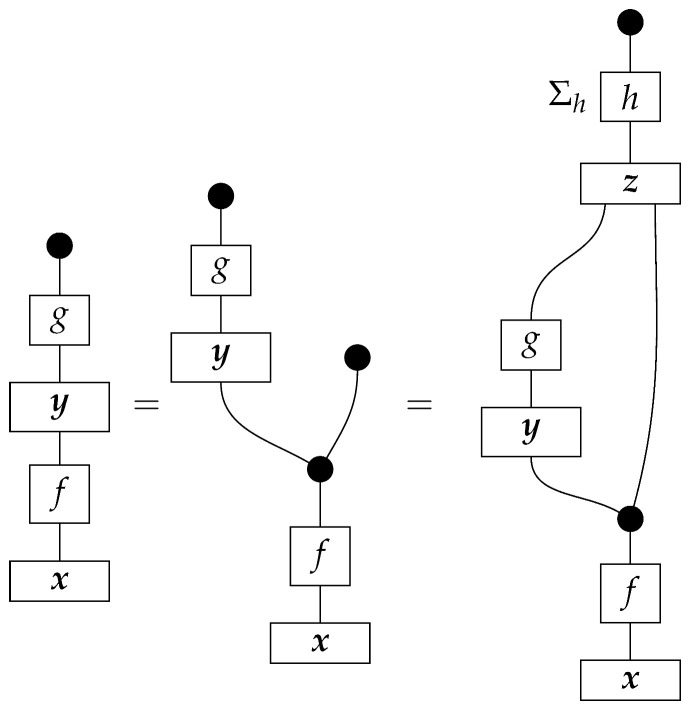

now with both *f* and *g* varying over marginally informationally complete sets but with *h* varying over the branches of only one instrument. The numbers on the left for various *f* and *g* determine y, because the assumption of marginal informational completeness of the sets {f} and {g} implies that the set of states f∘x is informationally complete for *X* (the input system-type of y) and the set of effects $d_{Y}\circ g$ is informationally complete for *Y* (the output system-type of y).

Now, consider the set of accessible probabilities

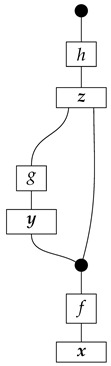

where *f*, *g*, and *h* vary over marginally informationally complete sets. Marginal informational completeness of {h} and {f} implies that the set of effects $d_{Z}\circ h$ and the set of states f∘x are informationally complete for *Z* and *X*, respectively. Thus, knowing these probabilities amounts (by tomographic locality) to knowing the value of the process

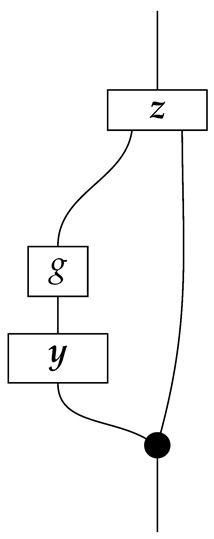

for each *g*. Therefore, for every pure (normalized) state

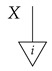

we can compute the quantity

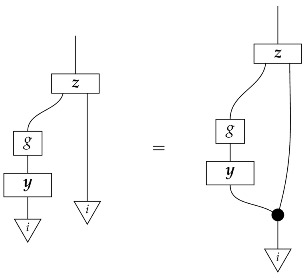

for each *g*. For each *i*, the state y∘i has full support due to the assumption that the model is strictly positive. Hence, by the assumed marginal informational completeness of {g}, varying *g* with fixed *i* yields an informationally complete set of states ρ=g∘y∘i. All in all, we now know a set of quantities

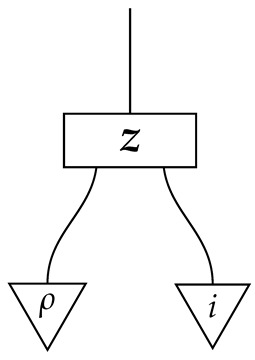

with ρ and *i* varying independently over informationally complete sets. This set of states determines the process z by tomographic locality.

**Proof of Theorem 1.**  If the given graph has *m* nodes, then the unknown model can be rewritten in a form without explicit common-cause maps:
(27)
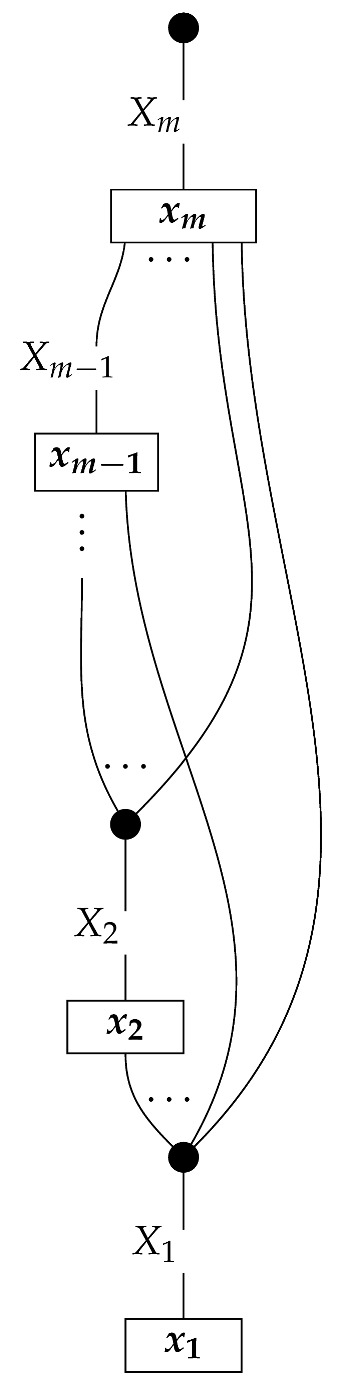

Here, the processes $x_{i}$ for *i* from 1 through *m* have been linearly ordered in correspondence with a topological ordering of the graph. The topological ordering also dictates the labeling of nodes in the graph and loci in the syntactic and semantic causal structures. The copy map for type $X_{i}$ and for *i* between 1 and *m* has m−i output wires, which connect as inputs to the boxes $x_{m-i}$ through $x_{m}$. Typically, the topological ordering does not have the property that every vertex is a parent (in the graph) of every vertex subsequent to it (in the ordering); however, there is no loss of generality in adding connections to the diagram of the unknown model. In effect, we declare that we might not make full use of the causal hypotheses encoded in the graph. For example, if node $X_{i}$ precedes $X_{j}$ in the topological ordering but there is no edge from $X_{i}$ to $X_{j}$ in the graph, then map $x_{j}$ in the syntactic causal structure has no input $X_{i}$, and similarly, the map $x_{j}$ in the original semantic causal structure has no such input. The semantic causal structure remains unchanged if we substitute for $x_{j}$ a map $x_{j}⊗d_{X_{i}}$ (composed with appropriate swap maps) that does have an input $X_{i}$. We make this kind of substitution everywhere it is possible, but retain the names of maps from the semantic causal structure for convenience.

The proof of identifiability constructs an iterative identification procedure, proceeding by double induction. Suppose that the value of $x_{i}:X_{1}⊗\ldots ⊗X_{i-1}\to X_{i}$ is known for all i<n. We show how to compute $x_{n}$.

For any locus $X_{k}$, let ${\left\{\phi_{k}\right\}}_{\phi }$ be the set of branches appearing in instruments in the accessible set $I_{X_{k}}$. Consider the set of processes
(28)
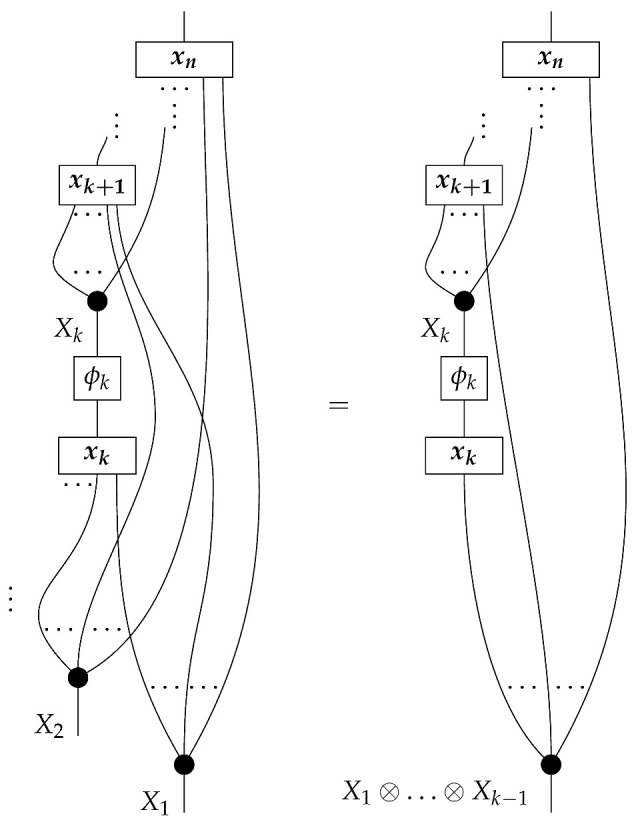

where, for all loci $X_{i}$ with *i* between *k* and n−1 (inclusive), all possible branches $\phi_{i}$ are plugged in such that the set of processes is indexed by $\phi_{k},\ldots ,\phi_{n-1}$. The left-hand diagram has inputs $X_{1}$ through $X_{k-1}$, each followed by a copy map with n−k+1 outputs connected to the boxes $x_{k}$ through $x_{n}$. The diagram has one output, from $x_{n}$.

We will show by induction that this set of processes can be computed for every *k* from 1 through *n*. In the base case, there are no input wires. Each locus from $X_{1}$ through $X_{n-1}$ is filled with an instrument branch. Each locus after $X_{n}$ is filled with each of the branches of one accessible instrument and the results are summed so that the discarding map following the final locus $X_{m}$ “falls through” every component process after $x_{n}$. For every such combination of branches, the resulting state on $X_{n}$ is tomographically determined by the results of various instruments at $X_{n}$; that is, for fixed $\phi_{1},\ldots ,\phi_{n-1}$, the state

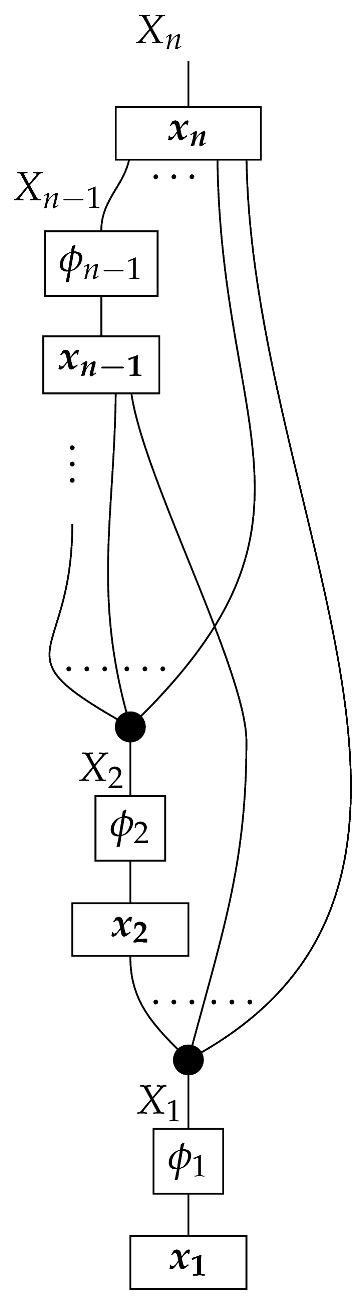

is determined by numbers

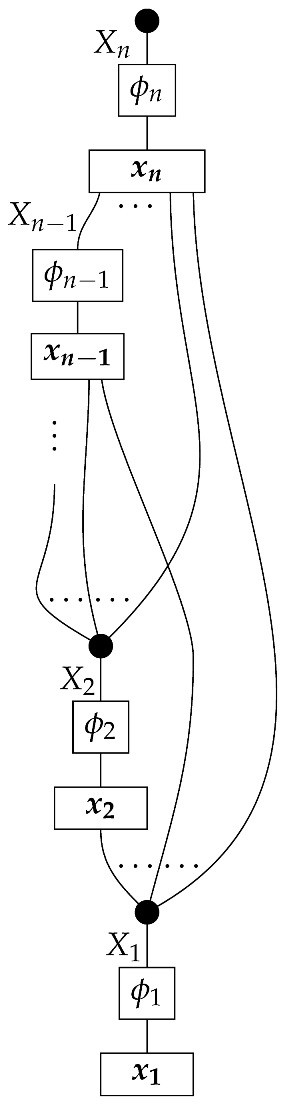

with $\phi_{n}$ varying, as marginal informational completeness of the set ${\left\{\phi_{k}\right\}}_{\phi }$ implies that the set of effects $d_{X_{n}}\circ \phi_{n}$ is informationally complete for $X_{n}$.

Now, suppose the set of processes (28) is known for some fixed *k*. We will show how to compute the set for k+1. For each $X_{i}$ with *i* between 1 and k−1 (inclusive), we introduce the set ${\left\{\epsilon_{i}\right\}}_{\epsilon }$ of states on $X_{i}$ (i.e., column vectors), each having entry 1 in exactly one position and 0s elsewhere. This set is informationally complete for $X_{i}$ (the set of product states $\left\{\epsilon_{1}⊗\ldots ⊗\epsilon_{k-1}\right\}$, where each ϵ varies independently, is informationally complete for $X_{1}⊗\ldots ⊗X_{k-1}$). Moreover, the states are copied by the copy map for $X_{i}$. We can plug an arbitrary $\epsilon_{i}$ into input wire $X_{i}$ for each *i* from 1 through k−1. The result is shown below.

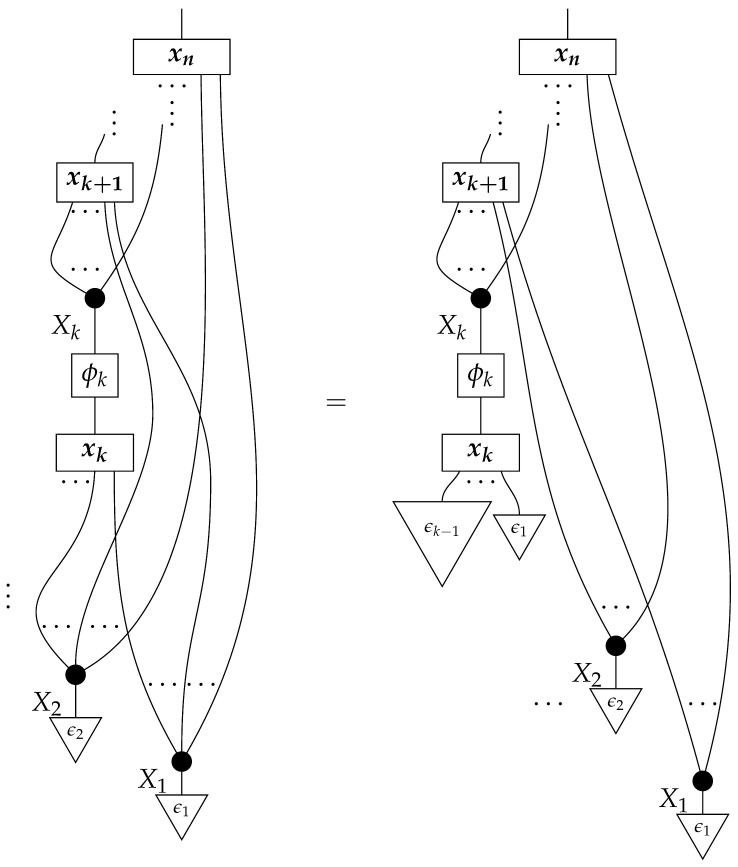

The copy maps on $X_{1}$ through $X_{k-1}$ in the right-hand diagram each have n−k outputs connected as inputs to boxes $x_{k+1}$ through $x_{n}$. The states $\epsilon_{i}$, as used here, have not been realized via local intervention regimes; rather, they are simply known processes for which composites with other known processes can be computed.

The state $x_{k}\circ \left(\epsilon_{k-1}⊗\ldots ⊗\epsilon_{1}\right)$ has full support per the standard strict positivity assumption about unknown models. Therefore, the set of states $\phi_{k}\circ x_{k}\circ \left(\epsilon_{k-1}⊗\ldots ⊗\epsilon_{1}\right)$ with ϕ varying is informationally complete for $X_{k}$, meaning that the process

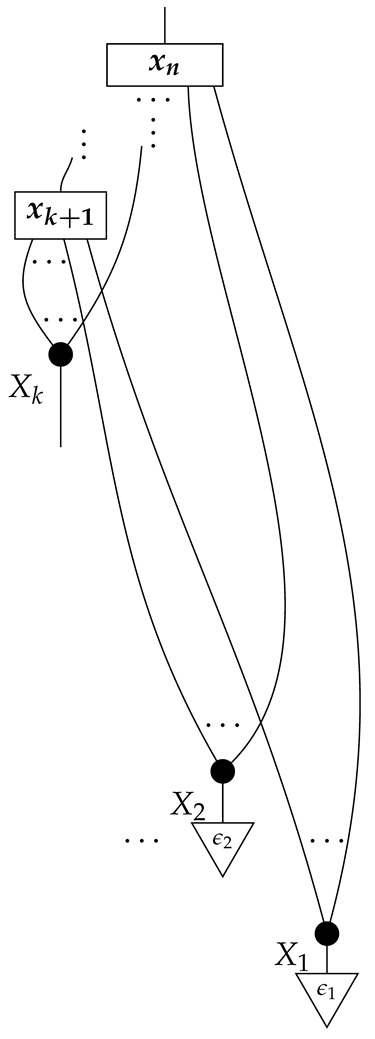

is known. Now, because the states $\epsilon_{1}$ through $\epsilon_{k-1}$ are arbitrary choices from the relevant sets of vectors, varying them yields knowledge of the following process.

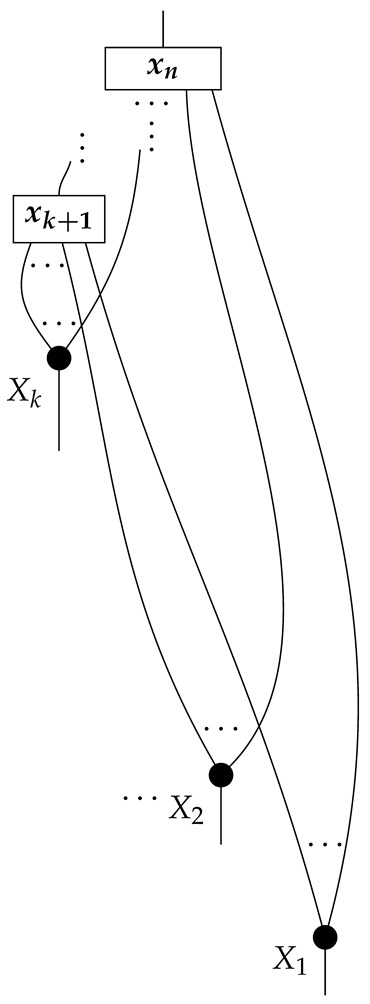

This completes the inner inductive step. Iterating eventually eliminates from the known process all boxes prior to $x_{n}$ while reducing each copy map’s number of outputs to one, making the final known process simply

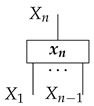

up to reordering of the input wires. Thus, the outer inductive step is completed. □

**Example 17.**  *The theorem guarantees that Markovian models based on the two-node graph in* (12) *with |X|=|Y|=2 are identifiable if the accessible set of instruments at X consists of the single instrument with branches ψ∘ϕ and $\psi^{\prime }\circ \phi^{\prime }$ in Example 14 and if the accessible set of instruments at Y consists of the same instrument. By Proposition 6, the accessible sets in this case are marginally informationally complete. The theorem also covers Example 14 itself, in which the instrument used at Y is a perfect passive observation instrument.*

**Example 18.**  *For the three-node graph in* (26) *with |X|=|Y|=2 and |Z|=n, where n is a positive integer, Markovian models are identifiable if the accessible set of instruments at X is the singleton consisting of the instrument in Example 15, the accessible set at Y is the singleton consisting of the instrument in Example 16, and the accessible set at Z is the singleton consisting of a perfect passive observation instrument n→n.*

## 6. Discussion

In this paper, we have presented a graphical causal modeling framework in which new and quite general kinds of causal identification problems can be posed. We have solved a problem only in the case of Markovian models, illustrating with this simple but important case the kind of reasoning our framework can support and the power of marginally informationally complete sets of instruments. Our main result raises the question of how much of the established theory of causal identification remains intact if we translate it to our framework and let the accessible sets of instruments be arbitrarily marginally informationally complete sets rather than singletons consisting of perfect passive observation instruments. Further investigation need not focus on marginally informationally complete sets of instruments, however; indeed, we can start with a causal model and ask what kinds of local intervention regimes would permit identification of various causal quantities.

Using the formalism in this paper also makes it possible to move beyond local intervention regimes and reason about intervention regimes in which the outcome of an intervention at one locus determines or influences the choice of instrument at another locus. Moreover, as indicated in Section 4.2, it is straightforward to modify the DAG-specific recipes in order to handle causal modeling based on other kinds of graphs that capture different kinds of qualitative hypotheses about the nature of latent variables.

Finally, many of the abstractions used in this paper are derived from quantum information science, and the ideas developed here may in turn be applied to the very active research area of “quantum causal modeling”. Initial studies of quantum causal identification with methods similar to those in this paper can be found in [8,9].

## Data Availability

No new data were created or analyzed in this study. Data sharing is not applicable to this article.
